# Supplementary material for: Single-cell analysis identifies conserved features of immune dysfunction in simulated microgravity and spaceflight
Source: Nat Commun. 2024 Jun 11;15:4795. doi: 10.1038/s41467-023-42013-y (PMC11166937; doi:10.1038/s41467-023-42013-y)
Supplement: Supplementary file 1 — Supplementary Information [file 41467_2023_42013_MOESM1_ESM.pdf]

Supplementary Information:

Single Cell Analysis Identifies Conserved Features of Immune Dysfunction in Simulated Microgravity and Spaceflight

Supplementary Figures S1-S21

Supplementary Data 1-19

Supplementary Figure 1 (related to Figure 1)

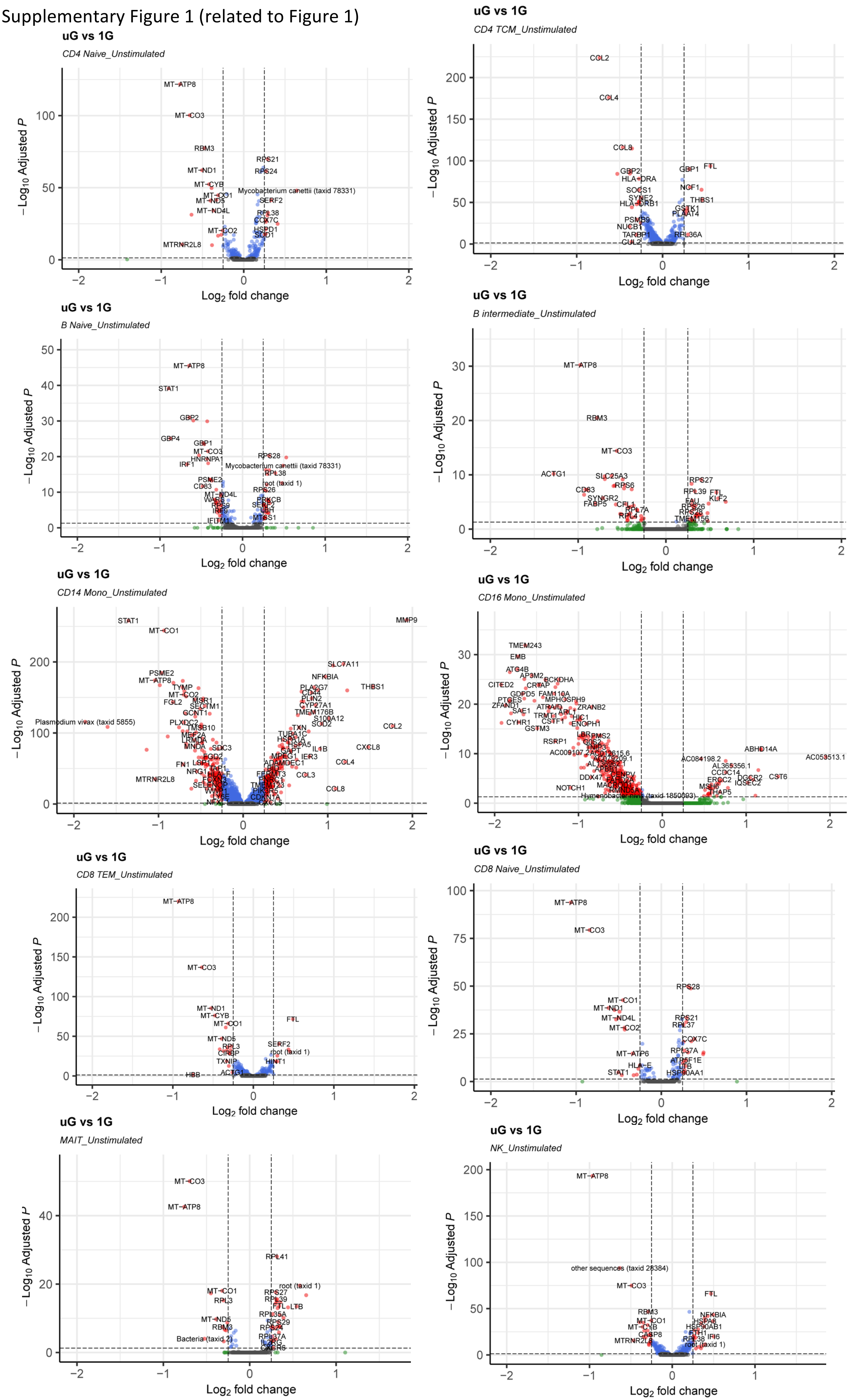

Supplementary Fig. 1: Volcano plots of differentially expressed genes (DEGs) between simulated microgravity (uG) and 1G (25 hours exposure) for 10 of the most abundant immune cell types. Benjamini-Hochberg (B-H) adjusted p-value (Adj.p) cutoff is 0.05, and log2Fold Change (log2FC) cutoff is 0.25.

Supplementary Figure 2 (related to Figure 2)

A

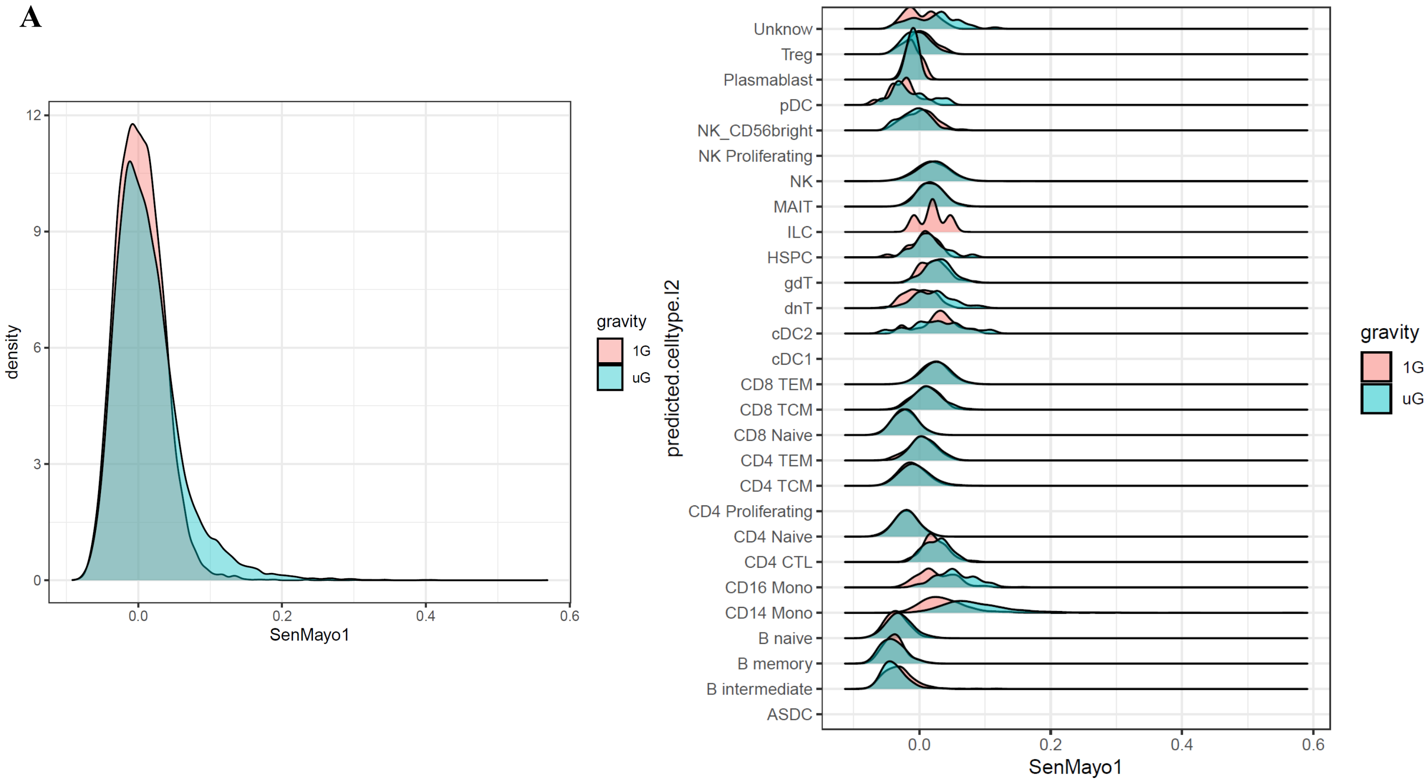

B

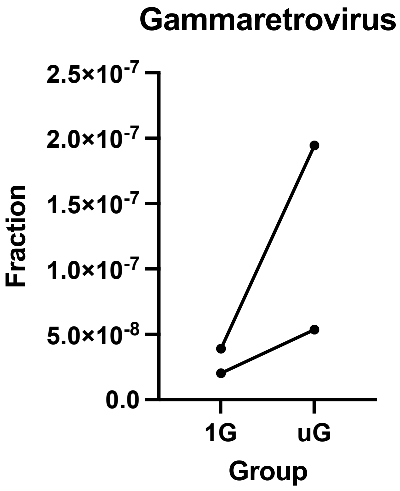

C

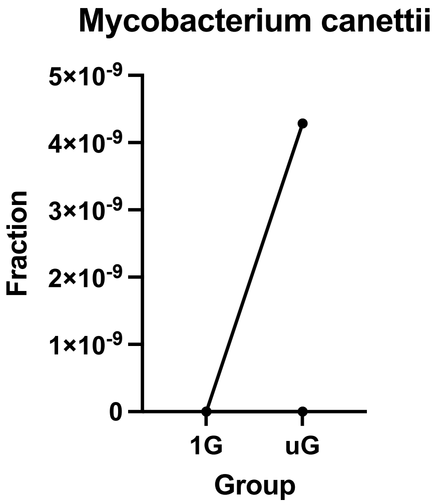

Supplementary Fig. 2: (A) Smooth density distribution of SenMayo scores in 1G and uG conditions (25 hours exposure) of total PBMCs (left) and individual cell type (right).
(B-C) Microbial gene expression validation analysis. Single-cell RNA-seq data of PBMCs from two donors (1 male, 1 female) were re-analyzed using alignment tool Magic-Blast to quantify the reads amount for *Gammaretrovirus* (B), and *Mycobacterium canettii* (C). The microbial quantity was represented by the fraction of the microbial reads to the total reads counts in the sample.

Supplementary Figure 3 (related to Figure 2)

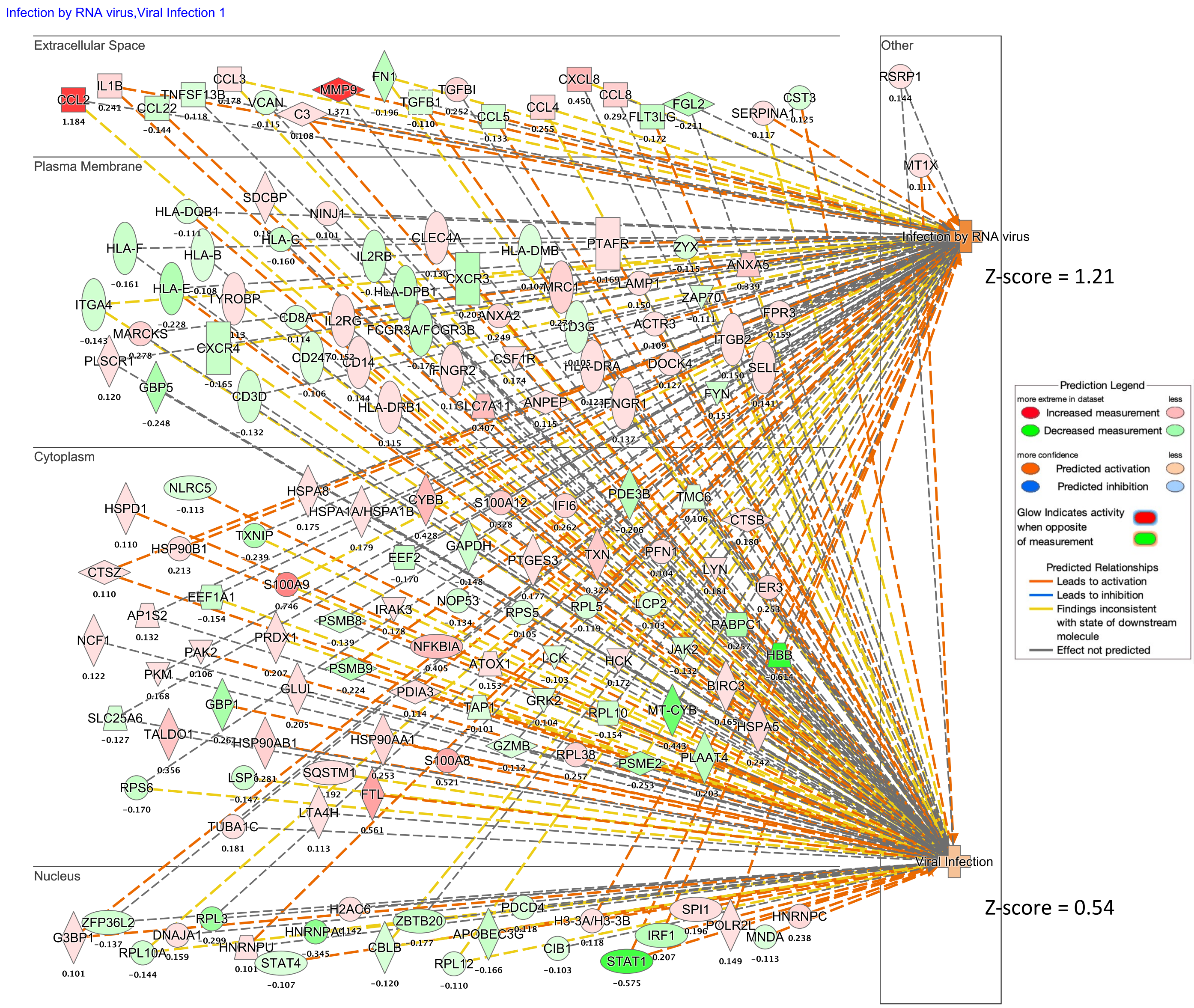

Supplementary Fig. 3: IPA functional analysis of 375 core genes in uG vs. 1G PBMCs. The biological functions of Infection by RNA virus and viral Infection are predicted to be upregulated, which is consistent with our viral abundance results in Fig2C. The log2FC of each gene related to its function is listed under the gene name. The enrichment p-value of viral infection and infection by RNA virus are 6E-44 and 1.03E-32, respectively.

Supplementary Figure 4 (related to Figure 2)

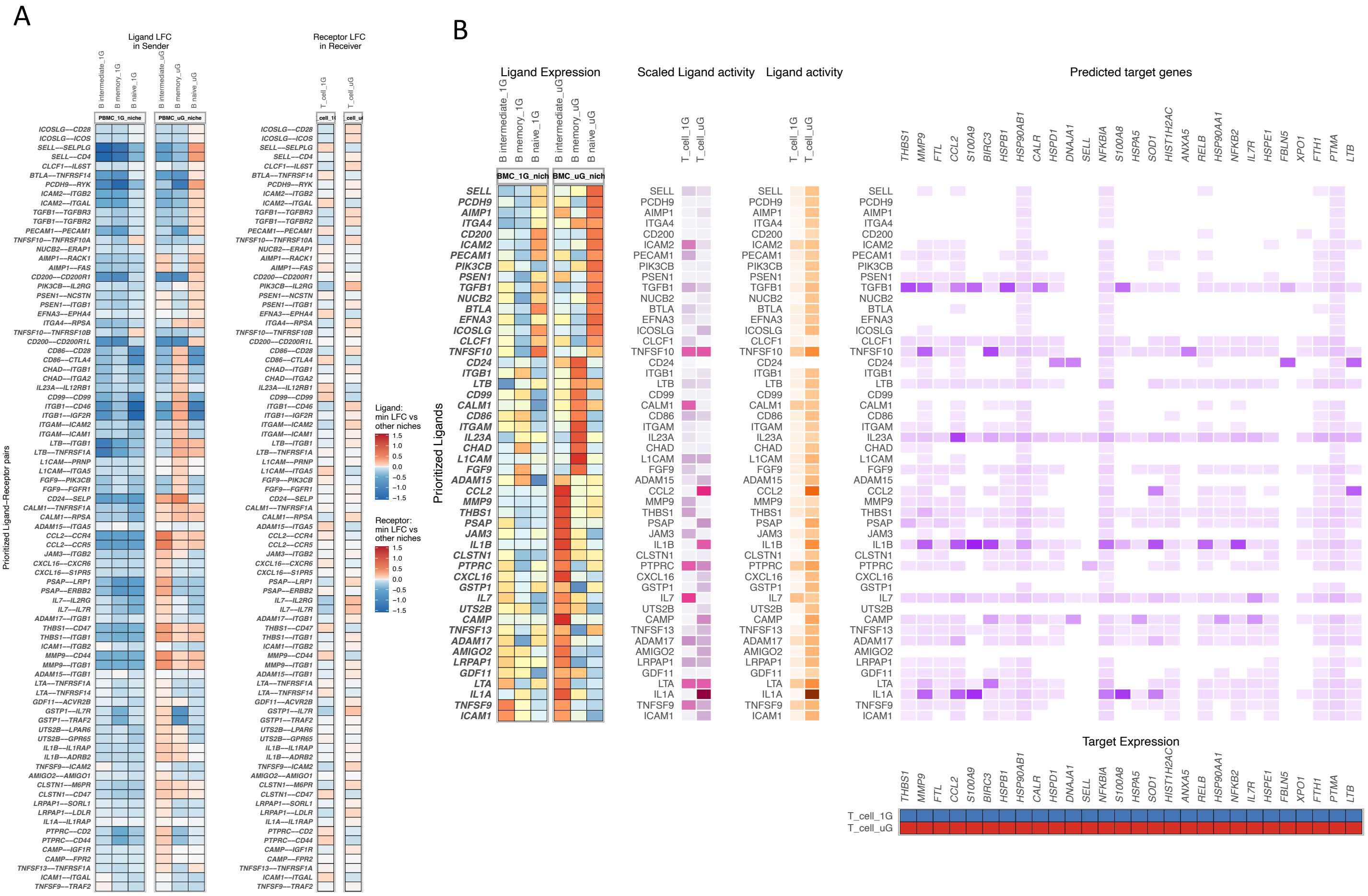

Supplementary Fig. 4: Differential NicheNet ligand-receptors analysis between uG vs. 1G conditions in unstimulated PBMCs. The sender cell type is the B cell, which provides ligands, whereas the receiver cell type is the T cell.

(A) Differential expression of ligand and receptor. First, the minimum log2FC of ligand gene expression in sender cells compared to all the sender cell types of the other niche is calculated, and the top 30 ligands are prioritized. Then the top 2 receptors are selected by the highest minimum log2FC of the receptor gene in receiver cells.

(B) From left to right are the ligand gene expression level, ligand activity, and its target genes in the receiver cells. The target gene expression levels in the receiver cell type are used to define the ligand activity.

Supplementary Figure 5 (related to Figure 2)

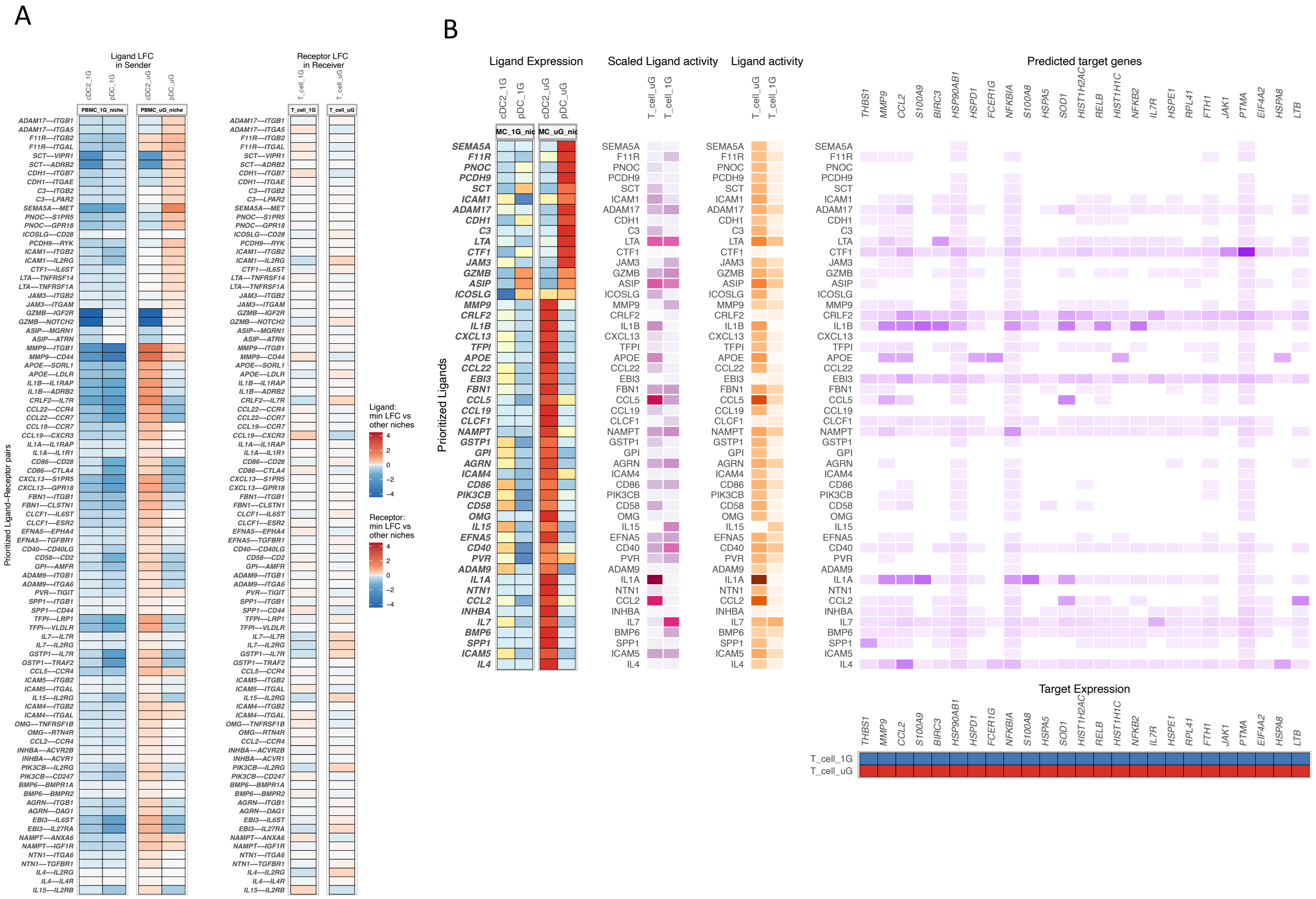

Supplementary Fig. 5: Differential NicheNet ligand-receptors analysis between uG vs. 1G conditions in unstimulated PBMCs. The sender cell type is the Dendritic Cell, which provides ligands, whereas the receiver cell type is the T cell.
(A) Differential expression of ligand and receptor. First, the minimum log2FC of ligand gene expression in sender cells compared to all the sender cell types of the other niche is calculated, and the top 30 ligands are prioritized. Then the top 2 receptors are selected by the highest minimum log2FC of the receptor gene in receiver cells.
(B) From left to right are the ligand gene expression level, ligand activity, and its target genes in the receiver cells. The target gene expression levels in the receiver cell type are used to define the ligand activity.

Supplementary Figure 6 (related to Figure 2)

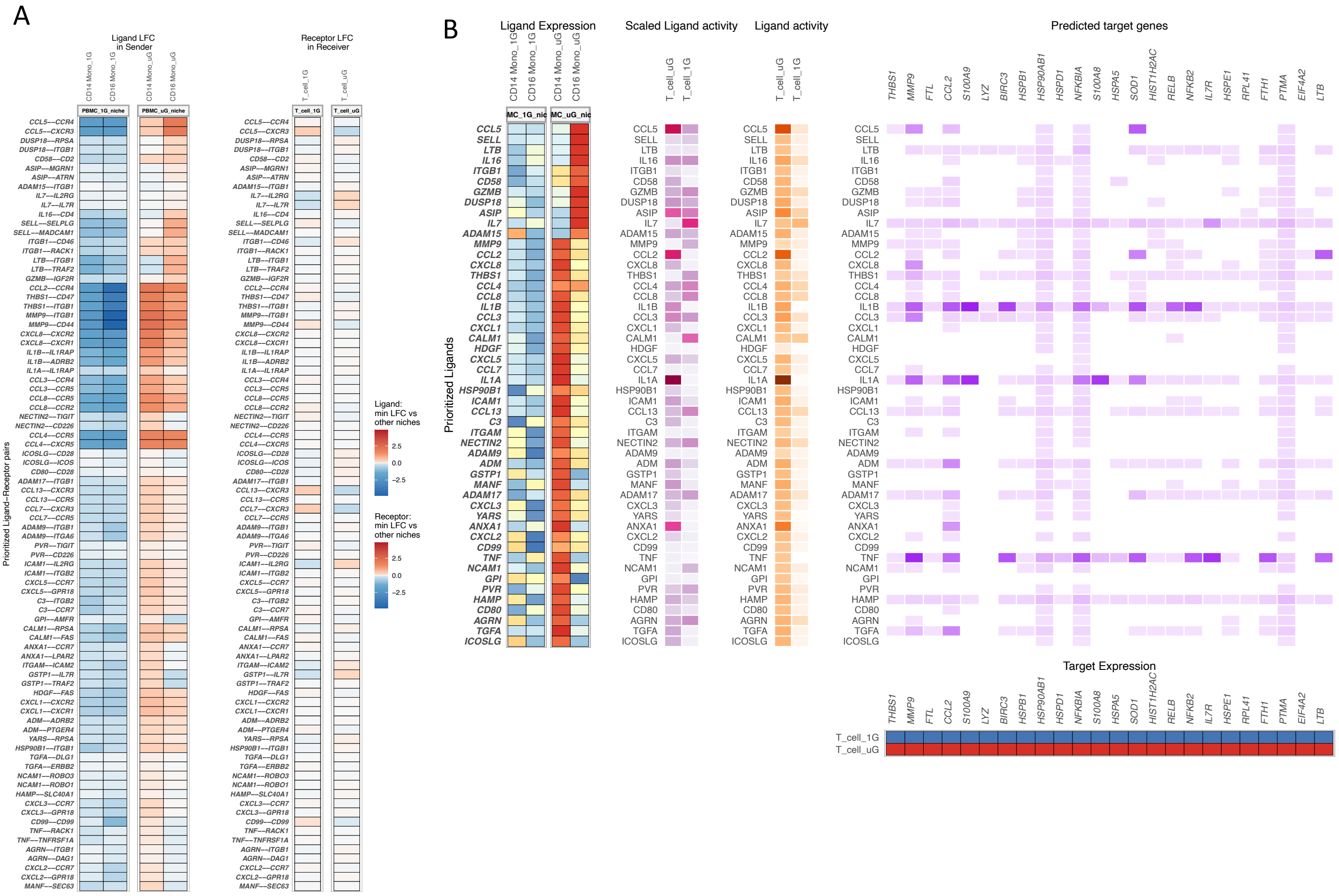

Supplementary Fig. 6: Differential NicheNet ligand-receptors analysis between uG vs. 1G conditions in unstimulated PBMCs. The sender cell type is the monocyte, which provides ligands, whereas the receiver cell type is the T cell.

(A) Differential expression of ligand and receptor. First, the minimum log2FC of ligand gene expression in sender cells compared to all the sender cell types of the other niche is calculated, and the top 30 ligands are prioritized. Then the top 2 receptors are selected by the highest minimum log2FC of the receptor gene in receiver cells.

(B) From left to right are the ligand gene expression level, ligand activity, and its target genes in the receiver cells. The target gene expression levels in the receiver cell type are used to define the ligand activity.

Supplementary Figure 7 (related to Figure 3)

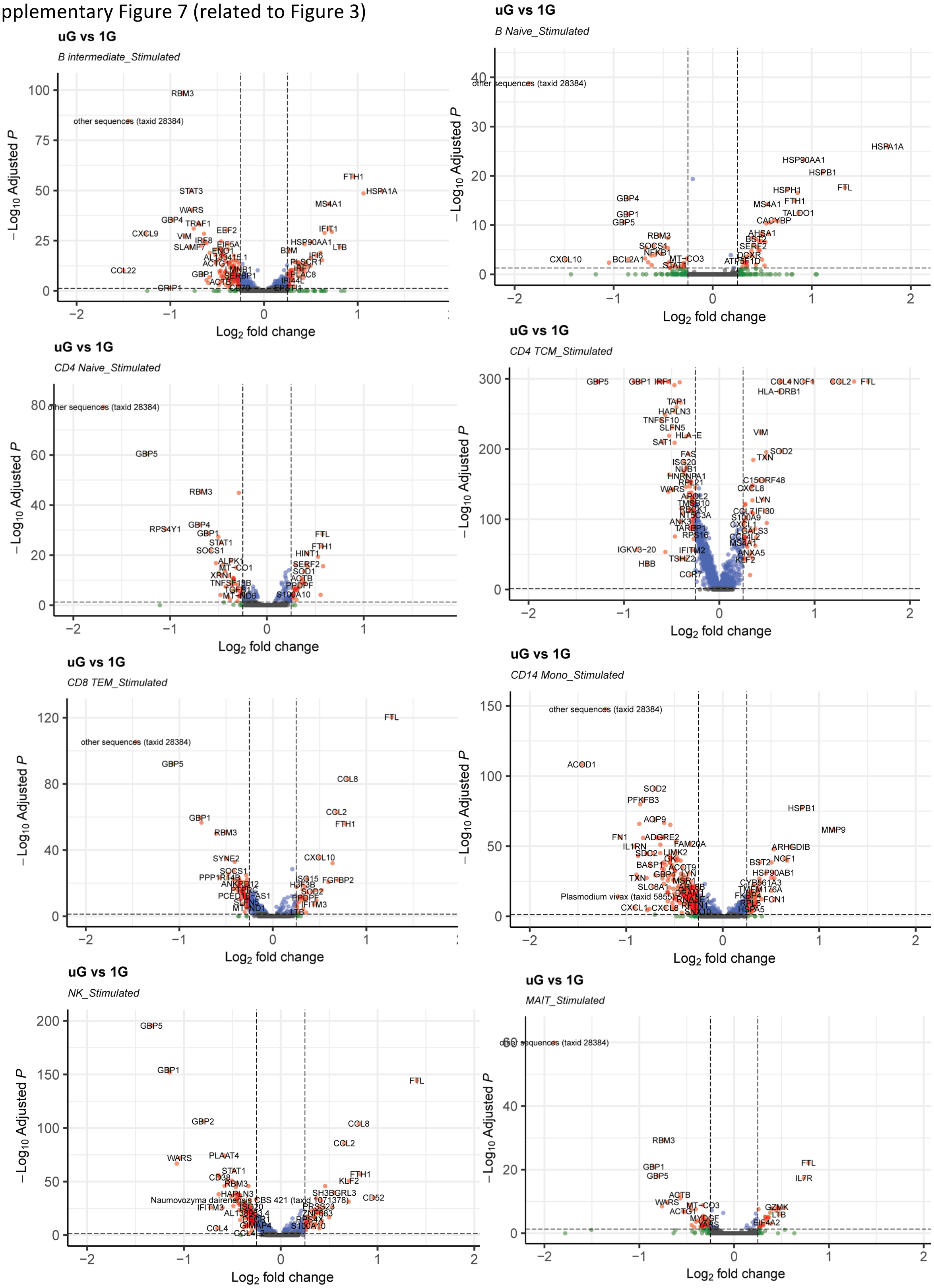

Supplementary Fig. 7: Volcano plots of differentially expressed genes (DEGs) between TLR7/8 stimulated (9 hours stimulation, 25 hours total culture) uG and 1G for the 8 most abundant immune cell types; B-H adj.p cutoff is 0.05, and log2FC cutoff is 0.25.

Supplementary Figure 8 (related to Figure 4)

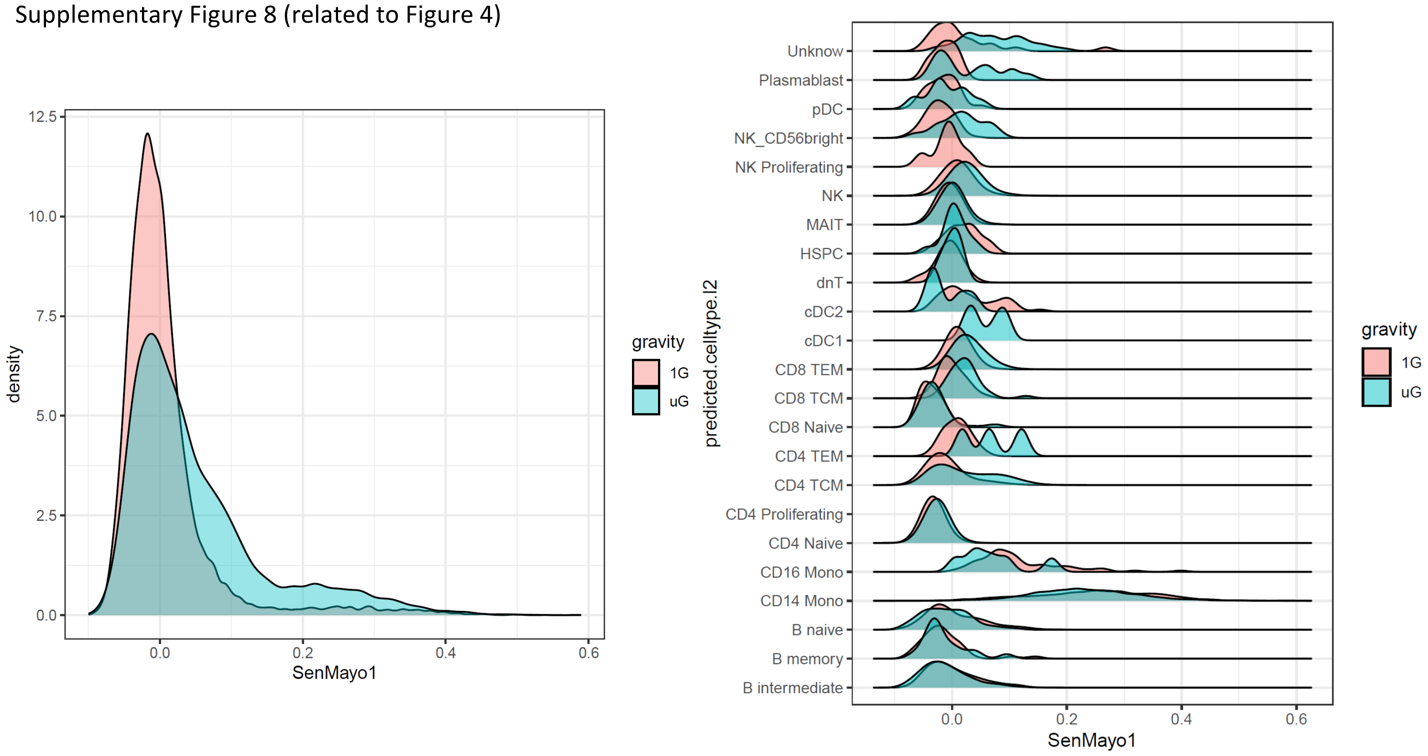

Supplementary Fig. 8: Smooth density distribution for SenMayo scores in TLR7/8 stimulated 1G and uG conditions stimulated (9 hours stimulation, 25 hours total culture) for general PBMCs (left) and individual cell types (right).

Supplementary Figure 9 (related to Figure 4)

A B

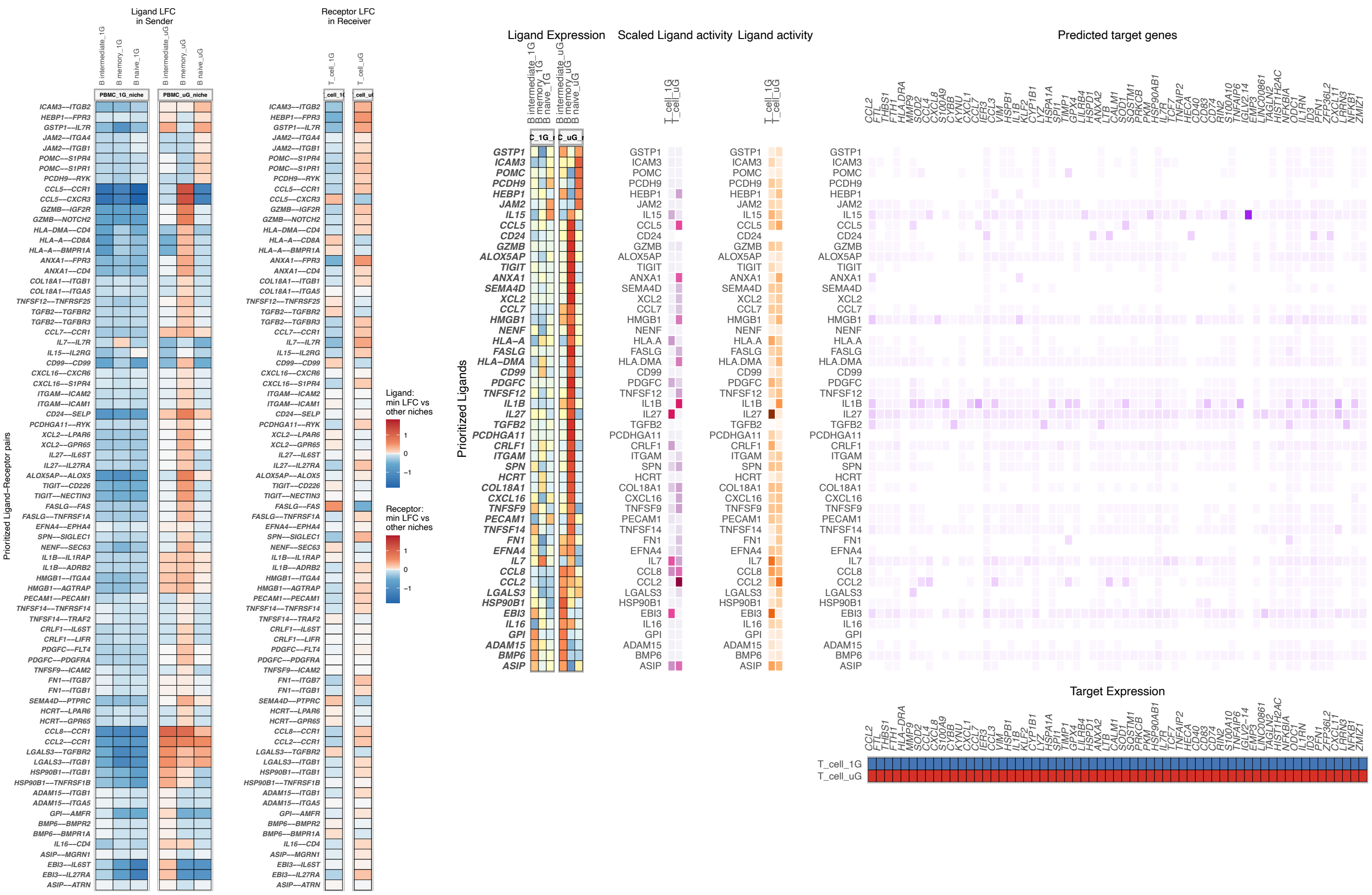

Supplementary Fig. 9: Differential NicheNet ligand-receptors analysis between uG vs. 1G conditions in stimulated PBMCs (9 hours stimulation, 25 hours total culture). The sender cell type that provides ligands is the B cell, and the receiver cell type is the T cell.

(A) Differential expression of ligand and receptor. First, the minimum log2FC of ligand gene expression in sender cells compared to all the sender cell types of the other niche is calculated, and the top 30 ligands are prioritized. Then the top 2 receptors are selected by the highest minimum log2FC of the receptor gene in receiver cells.

(B) From left to right are the ligand gene expression level, ligand activity, and its target genes in the receiver cells. The target gene expression levels in the receiver cell type are used to define the ligand activity.

Supplementary Figure 10 (related to Figure 4)

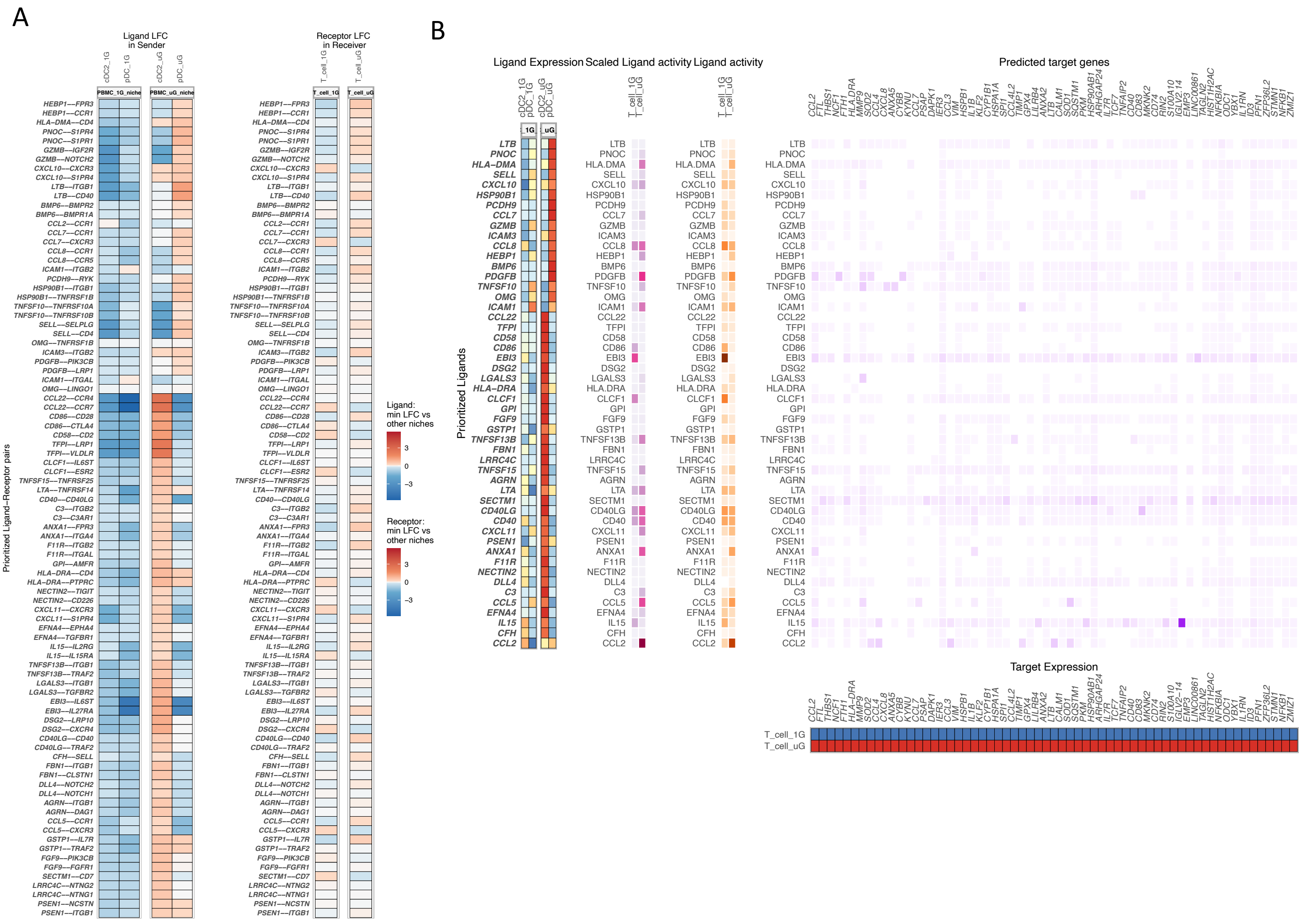

Supplementary Fig. 10: Differential NicheNet ligand-receptors analysis between uG vs. 1G conditions in stimulated PBMCs (9 hours stimulation, 25 hours total culture) . The sender cell type that provides ligands is the DC cell, and the receiver cell type is the T cell.

(A) Differential expression of ligand and receptor. First, the minimum log2FC of ligand gene expression in sender cells compared to all the sender cell types of the other niche is calculated, and the top 30 ligands are prioritized. Then the top 2 receptors are selected by the highest minimum log2FC of the receptor gene in receiver cells.

(B) From left to right are the ligand gene expression level, ligand activity, and its target genes in the receiver cells. The target gene expression levels in the receiver cell type are used to define the ligand activity.

Supplementary Figure 11 (related to Figure 4)

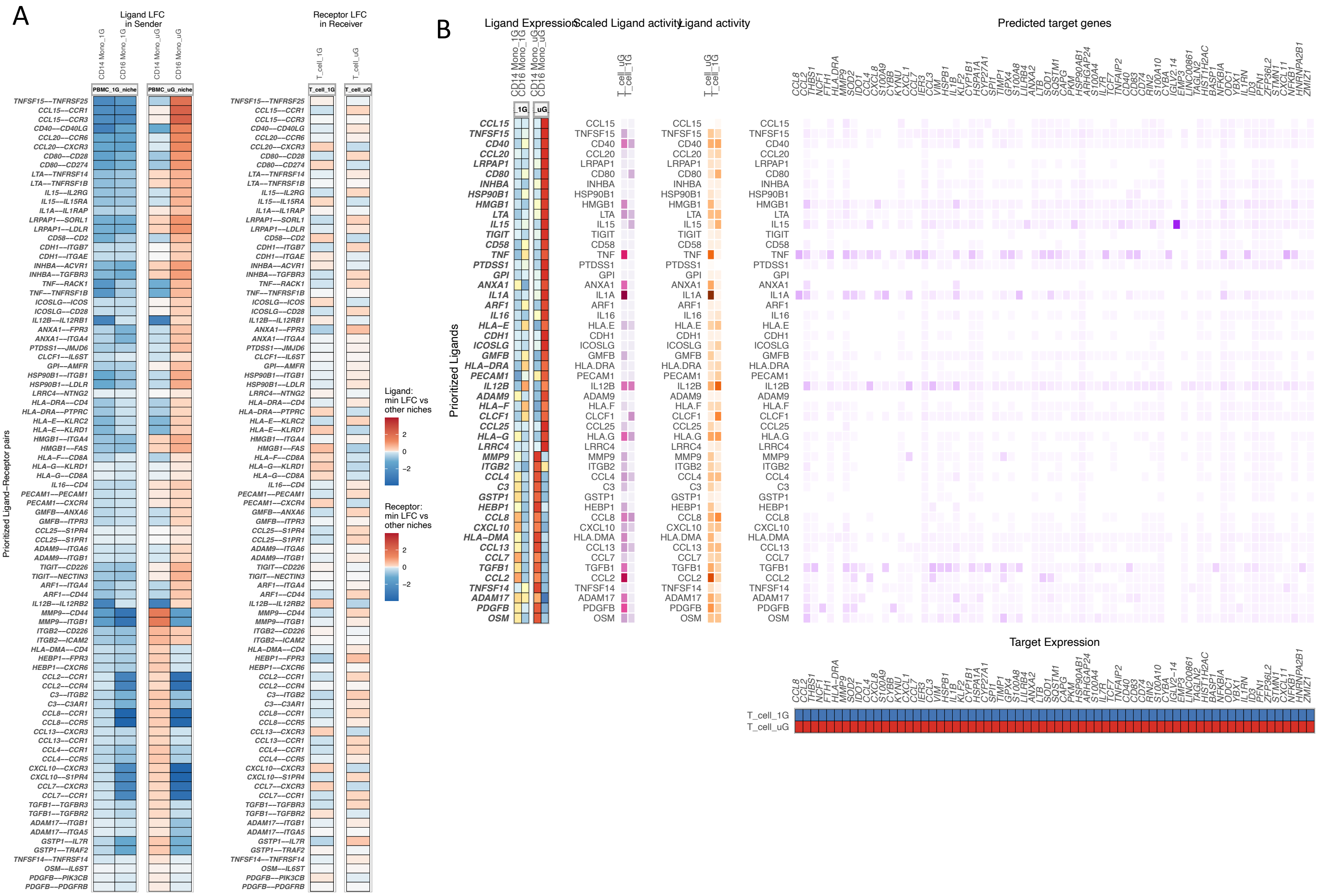

Supplementary Fig. 11: Differential NicheNet ligand-receptors analysis between uG vs. 1G conditions in stimulated PBMCs (9 hours stimulation, 25 hours total culture). The sender cell type that provides ligands is the monocyte, and the receiver cell type is the T cell.

(A) Differential expression of ligand and receptor. First, the minimum log2FC of ligand gene expression in sender cells compared to all the sender cell types of the other niche is calculated, and the top 30 ligands are prioritized. Then the top 2 receptors are selected by the highest minimum log2FC of the receptor gene in receiver cells.

(B) From left to right are the ligand gene expression level, ligand activity, and its target genes in the receiver cells. The target gene expression levels in the receiver cell type are used to define the ligand activity.

Supplementary Figure 12

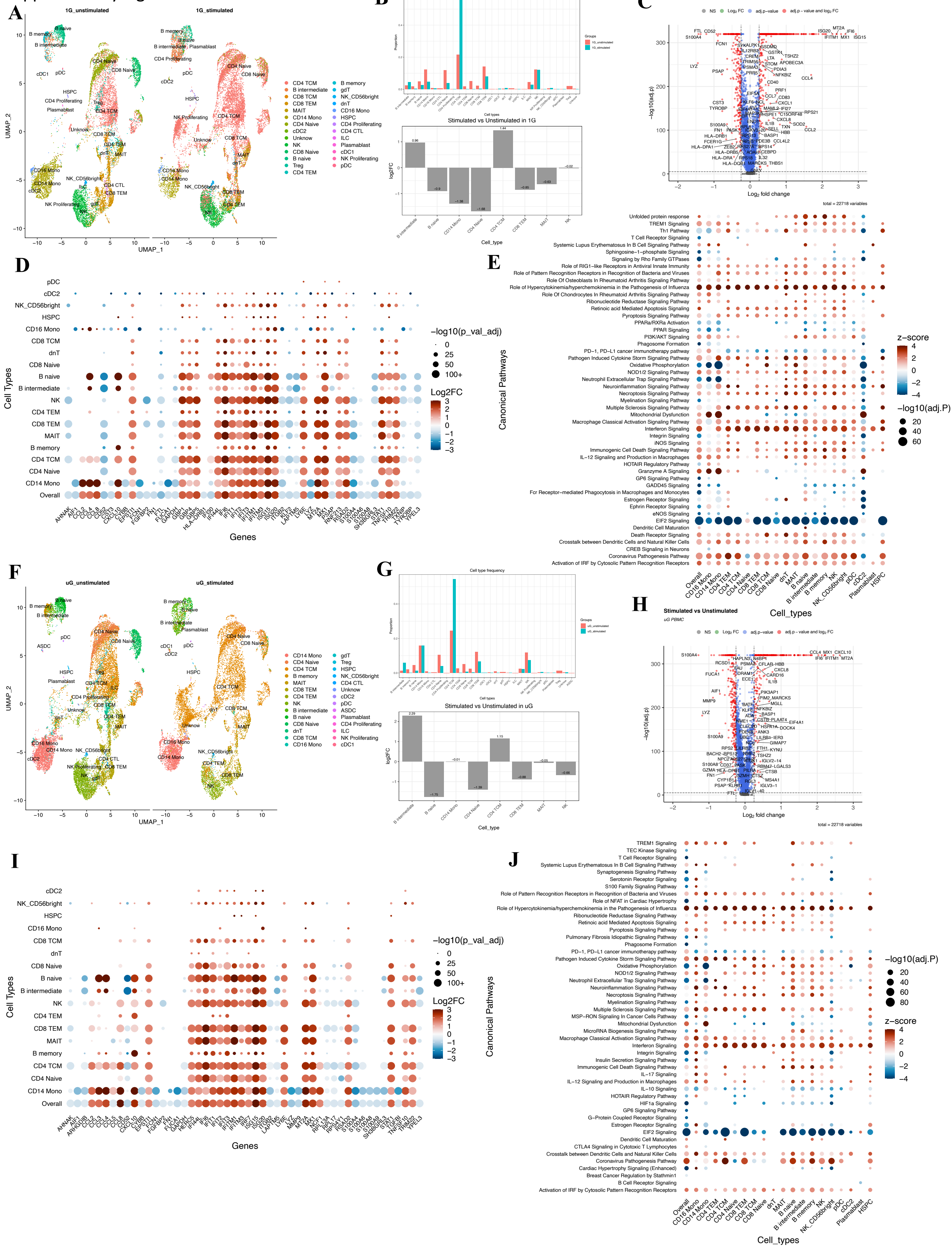

Supplementary Figure 12: Pattern recognition receptor stimulation alters the single-cell transcriptional landscape of human PBMCs in 1G and uG conditions.

(A-E) Stimulation alters the single-cell transcriptional landscape of human PBMCs in 1G**.**

(A) UMAP plot of unstimulated and R848 stimulated (9 hours) PBMCs single-cell transcriptomes (10X Genomics). Singe-cell RNA-seq data was integrated together from a male (36 years old) and a female (25 years old) donor, that underwent 1G for 25 hours. Cells were resolved into 27 distinct clusters.

(B) Quantification of relative abundance of each cluster of PBMCs by percentage, or log2FC between unstimulated and stimulated conditions under 1G. Source data are provided with the paper.

(C) Volcano plot of differentially expressed genes (DEGs) across all immune cell types between stimulated and unstimulated conditions under 1G; B-H adjusted p-value (adj. p) cutoff is 0.05, and log2FC cutoff is 0.25.

(D) Dot plot showing the top 25 most upregulated and top 25 most downregulated DEGs from (C) and their expression levels across 18 immune cell populations. Spot color reflects log2FC of stimulated vs unstimulated PBMCs under 1G, while spot size shows -log10 (adj. p).

(E) Canonical pathway enrichment analysis obtained from Ingenuity Pathway Analysis (IPA) is shown across 19 immune cell clusters. Spot color reflects IPA z-score enrichment of stimulated vs unstimulated PBMCs under 1G. Red shows predicted activation of the pathway in stimulated PBMCs and blue shows repression of the pathway in stimulated PBMCs. Spot size shows the level of significance via -log10 (adj. p).

(F-J) Stimulation alters the single-cell transcriptional landscape of human PBMCs in uG**.**

(F) UMAP plot of unstimulated and stimulated (R848, 9 hours) PBMCs single-cell transcriptomes. Singe-cell RNA-seq data was integrated from the same male and female donors, treated with uG condition for 25 hours. Cells were resolved into 28 distinct clusters.

(G) Quantification of relative abundance of each cluster of PBMCs by percentage, or log2FC between unstimulated and stimulated conditions under uG. Source data are provided with the paper.

(H) Volcano plot of DEGs across all immune cell types between unstimulated and stimulated conditions under uG; B-H adj. p cutoff is 0.05, and log2FC cutoff is 0.25.

(I) Dot plot showing the top 25 most upregulated and top 25 most downregulated DEGs from (H) and their expression levels across 17 immune cell populations. Spot color reflects log2FC of stimulated vs unstimulated PBMCs under uG, while spot size shows -log10 (adj. p).

(J) Canonical pathway enrichment analysis obtained from IPA is shown across 19 immune cell clusters. Spot color reflects IPA z-score enrichment of stimulated vs unstimulated PBMCs under uG, with red indicating predicted activation of the pathway in stimulated PBMCs and blue meaning repression of the pathway in stimulated PBMCs. Spot size shows the level of significance via -log10 (adj. p).

Supplementary Figure 13

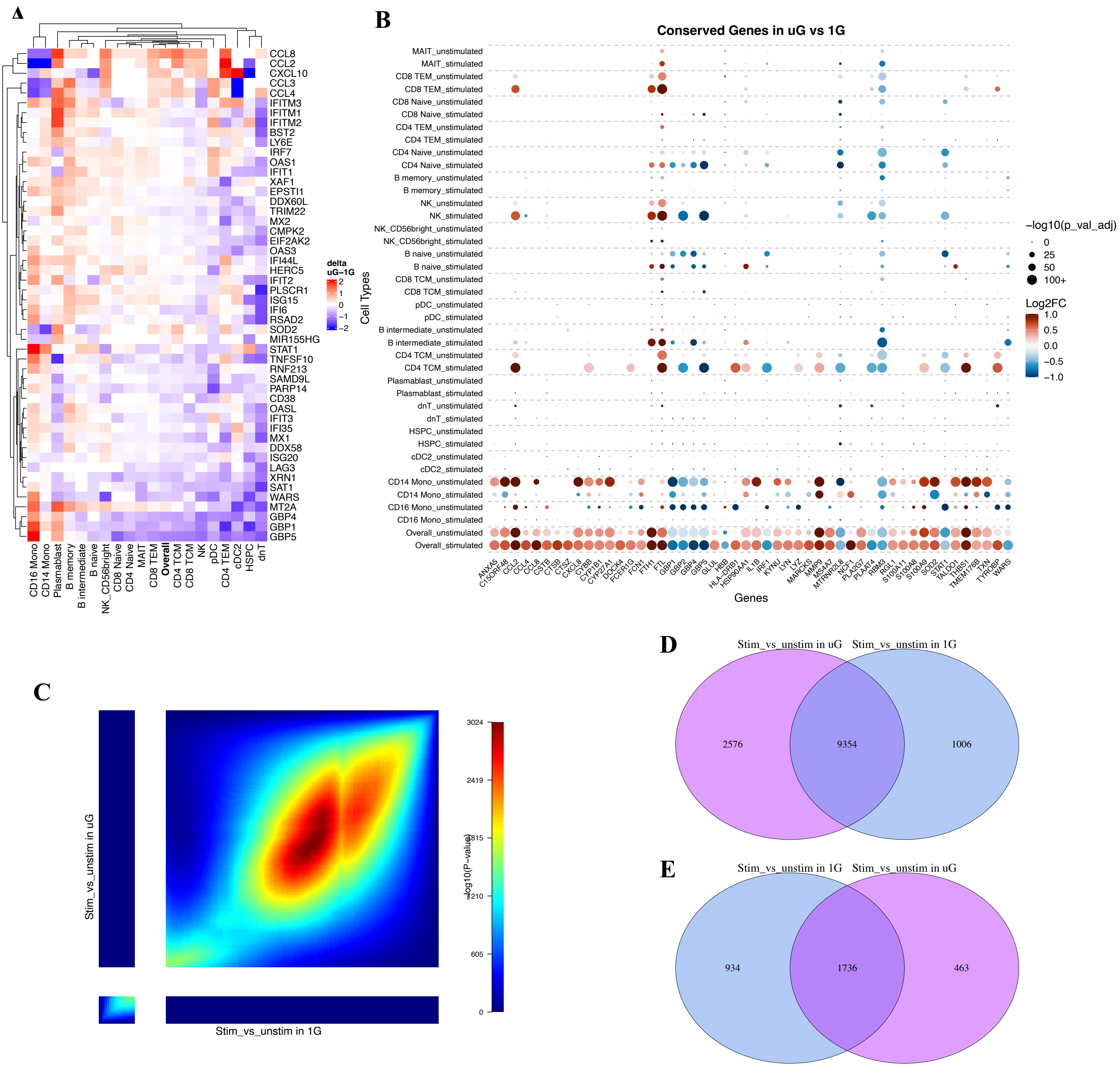

Supplementary Figure 13: Comparison of stimulated and unstimulated PBMCs under uG and 1G conditions.

(A) Comparison of log2FC in DEGs between stimulated and unstimulated PBMCs under uG and 1G conditions, presented as the difference in log2FC values (uG - 1G). The top 50 most upregulated DEGs between stimulated and unstimulated PBMCs under 1G were selected for comparison.

(B) Top 50 conserved DEGs specifically sensitive to uG, ranked by the absolute sum of log2FC values, derived separately from the sum of positive log2FC values and the sum of negative log2FC values, under both stimulated and unstimulated conditions in the "Overall" group, and with expression patterns displayed for all cell types.

(C) Rank-Rank Hypergeometric Overlap (RRHO) analysis shows the overlap in gene expression data between 1G and uG of stimulated vs unstimulated PBMCs. The x-axis and y-axis represent the ranks of the genes in the two gene lists, which were determined by calculating -log10(adj.p)*log2FC. The color represents the -log10 transform of the P-value, which was calculated using the hypergeometric test for each pair of ranks from the two ranked gene lists. Genes significantly changing in the same direction in both experiments are in the upper-right quadrant (both down) and bottom-left (both up) and in opposite directions in the upper-left and bottom-right.

(D-E) Venn diagram summarizes the overlapping of down-regulated (D) and up-regulated (E) genes of stimulated vs unstimulated PBMCs between 1G and uG conditions.

Supplementary Figure 14

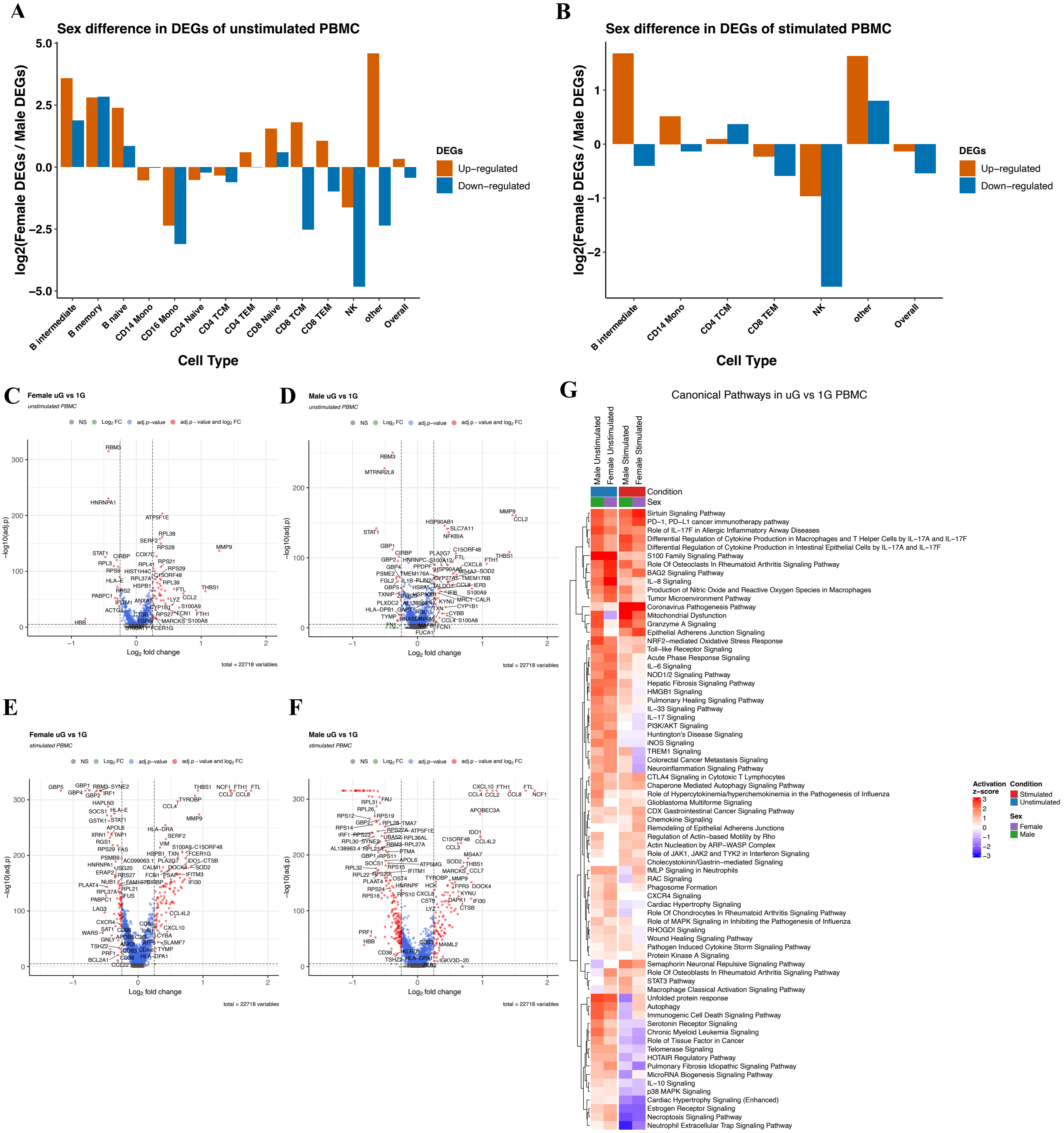

Supplementary Figure 14: Sex difference in response to simulated microgravity.

(A-B) Sex difference in the number of up-regulated and down-regulated DEGs in uG vs 1G of unstimulated (A) and stimulated (B) PBMCs. The comparison was presented by log2 fold change of the number of DEGs in female divided by the number of DEGs in male. The DEGs were determined by B-H adjusted P value <0.05 and the absolute log2FC>=0.1.

(C-D) Volcano plot of DEGs in uG vs 1G in unstimulated PBMC from female (C) and male (D) donor. B-H adj.p cutoff is 0.05, and log2FC cutoff is 0.25.

(E-F) Volcano plot of DEGs in uG vs 1G in stimulated PBMC from female (E) and male (F) donor. B-H adj.p cutoff is 0.05, and log2FC cutoff is 0.25.

(G) Comparison of IPA canonical pathways between the male and female samples. The pathways that are significantly (B-H adj.p<0.05) enriched in both sexes in each condition are shown on the heatmap.

Supplementary Figure 15 (related to Figure 5)

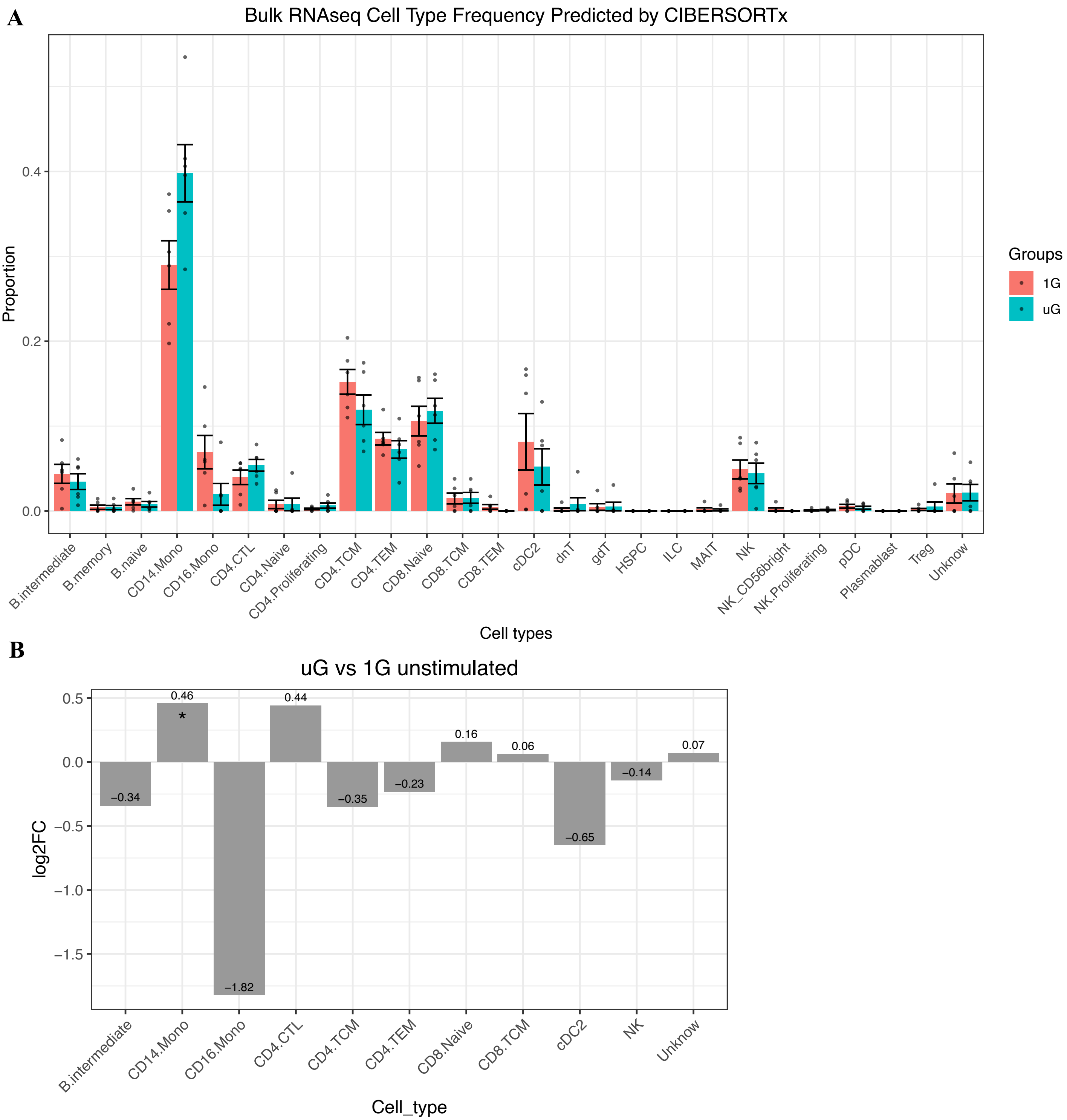

Supplementary Figure 15: Cell Type Frequency Changes within PBMCs as predicted by CIBERSORTx using Bulk RNA-seq.
(A) Bulk RNA-seq data of PBMCs from 6 donors (3 male, 3 female) in Fig. 5A were analyzed using CIBERSORTx to predict cell type frequency in the sample. Our single-cell RNA-seq data from PBMCs was used to build the Signature Matrix File as the reference to predict the cell proportion in the bulk RNA-seq data. The bar height represents the average proportion of the cell type in the group. The error bar shows the standard error. Dot represents the cell frequency in each sample. Source data are provided with this paper.
(B) Quantification of cell proportion alteration between uG and 1G in PBMC by log2Fold Change. The comparison was made by the Student's t-test, two-tailed. *p ≤ 0.05

Supplementary Figure 16 (related to Figure 5)

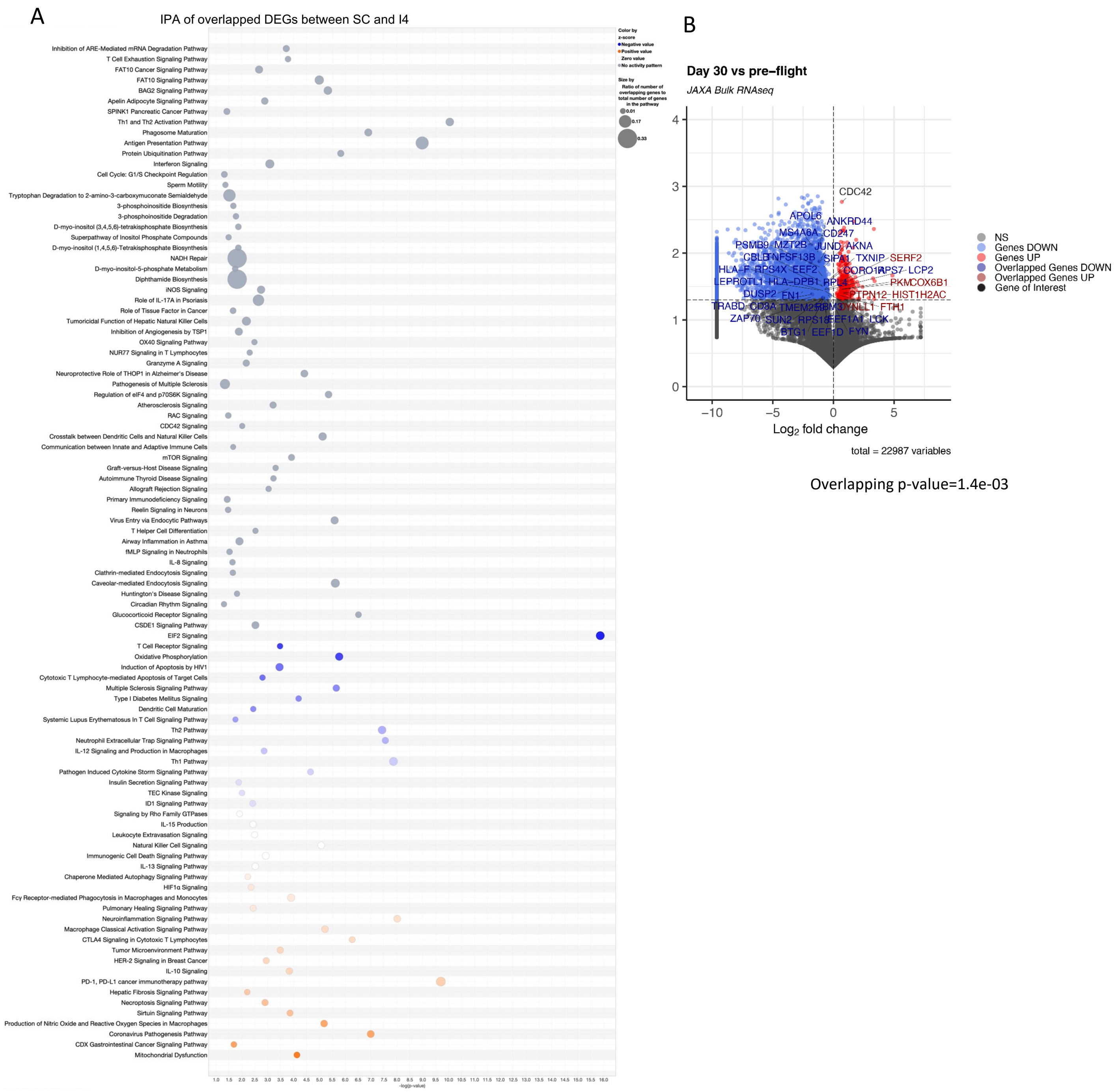

Supplementary Fig. 16: Single-cell transcriptomic signature validation with I4 and JAXA6 datasets.

(A) Pathway enrichment analysis of overlapped DEGs between human PBMCs(SC) and i4 (n=4) data. The shared DEGs between SC uG vs 1G and I4 post-flight (R+1) vs pre-flight (L-44) were further filtered by their directionality, and 122 altered genes with the same directions between SC and I4 were used for IPA analysis. Red indicates a predicted activation in pathways, blue indicates a predicted inhibition in pathways, and gray indicates pathways enriched but no prediction for activation or inhibition.

(B) Overlapping DEGs from uG vs. 1G between human PBMCs (single-cell core 375 gene list) and JAXA6 dataset (cell-free RNA 30 days in-flight vs pre-flight, n=6). Genes that are consistently up-regulated across single-cell and bulk sequencing are labeled in dark red; genes that are consistently down-regulated across single-cell and bulk RNA-seq are labeled in blue. One gene of interest, *Cdc42*, the most significantly upregulated gene after 30 days in-flight is listed in black. Genes that are not consistent across the two datasets are not labeled. The overlapping p-value was calculated by the Fisher's exact test, two-tailed.

Supplementary Figure 17

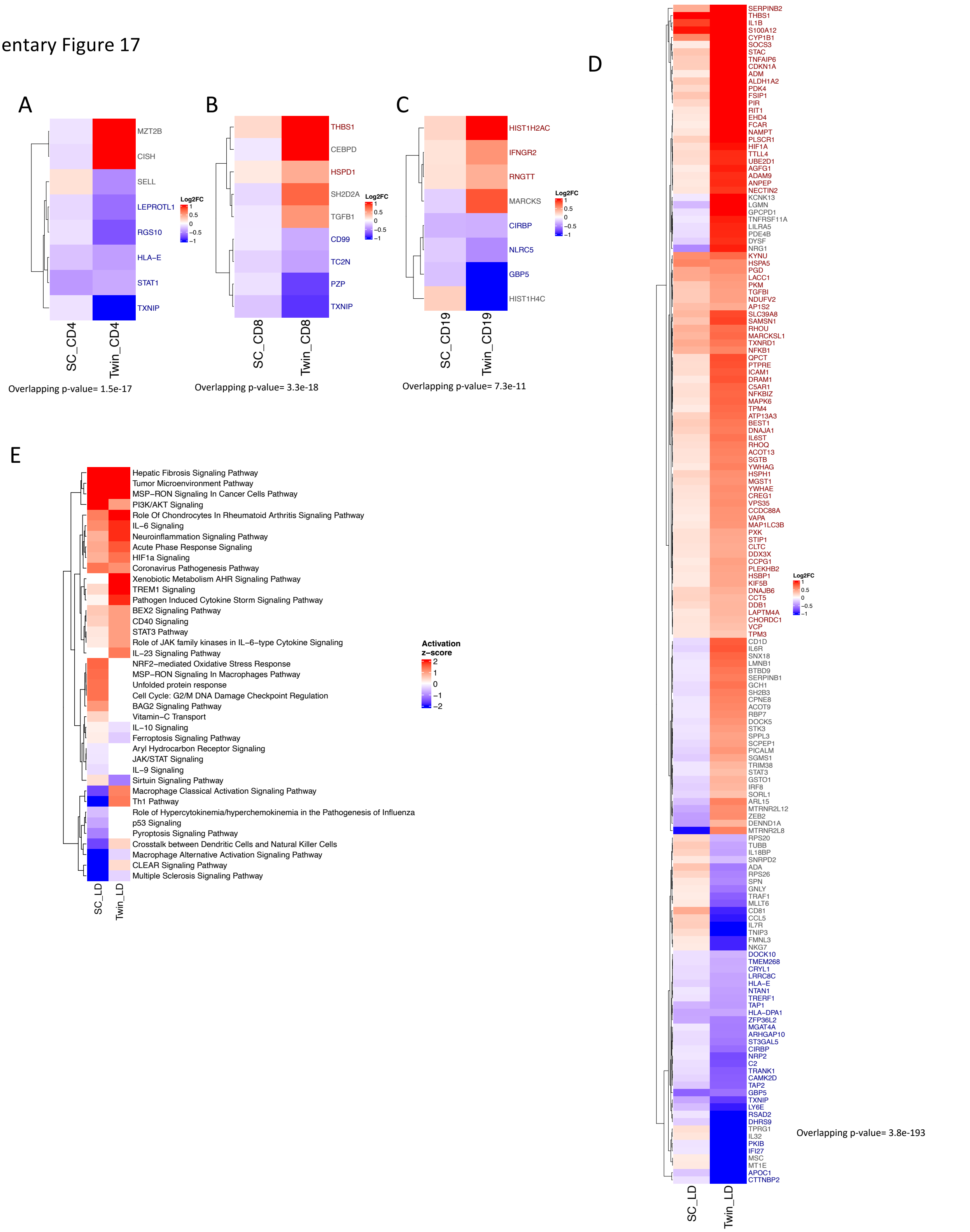

Supplementary Fig. 17: Single-cell transcriptomic signature validation employing data from the NASA Twins study. (A-D) Overlapping DEGs from uG vs. 1G between human PBMCs and the Twins datasets for CD4, CD8, CD19, and lymphocyte-depleted (LD) cell types. Both datasets are RNA-seq with DEGs defined by adjusted P-value <0.05 and log2FC>|0.1|. Genes that are consistently up-regulated across single-cell human PBMCs and the Twins datasets are labeled in dark red; genes that are consistently down-regulated across the two are labeled in dark blue. Genes that overlap, but are inconsistent in their expression directions are labeled in gray. One astronaut and his twin sibling participated in the study. The overlapping p-value was calculated by the Fisher's exact test, two-tailed.
(E) Pathway enrichment analysis of overlapped DEGs in LD cells between human PBMCs (single-cell) and the Twins study. Pathway enrichment result is only shown for LD fraction due to the abundance of overlapping genes.

Supplementary Figure 18 (related to Figure 6)

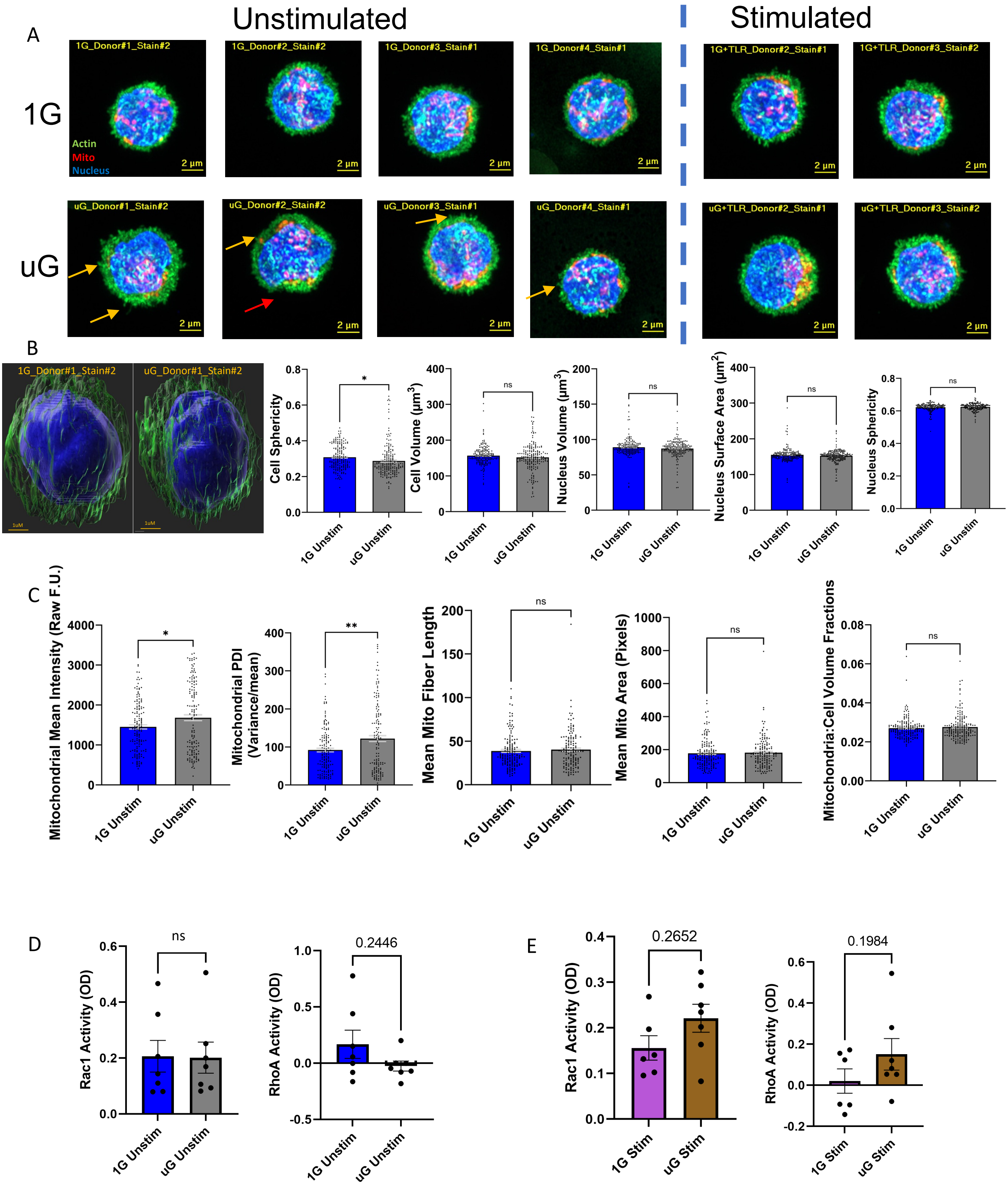

Supplementary Fig. 18: Cytoskeleton and mitochondrial assessment of PBMCs in simulated uG and 1G.
(A) Representative super-resolution microscopy images of PBMCs from 1G and uG from all 4 donors assessed. Arrows (orange) point to actin projections. Note border irregularity in some uG cells (red arrow). Scale bar=2um.
(B) 3D imaging, cell sphericity, cell volume, nucleus volume, nucleus surface area, and nucleus sphericity measured by super-resolution microscopy (25 hours, unstimulated). Dots represent parameters of individual PBMCs from 4 donors, 194 cells were imaged in 1G and 179 cells were imaged in uG. Welch's t test is used to calculate P values, $*P \leq 0.05$. Data are plotted as mean ± SEM and source data are provided with the paper.
(C) Two-dimensional mitochondrial maximum intensity projection analysis of mean MitoTracker Red intensity, mitochondrial Punctate Diffuse Index PDI (variance/mean), mitochondrial fiber length, mean mitochondrial area and volume fraction between 25 hours of 1G or simulated uG. Dots represent parameters of individual PBMCs (1G n=150 cells, uG n=142 cells) from 4 independent donors. Donors are male (25 years old), and females (35, 38 and 46 years old). Welch's t test is used to calculate P values, $**P \leq 0.01$, $*P \leq 0.05$. Data are plotted as mean ± SEM and source data are provided with the paper.
(D) G-LISA levels of active GTP-bound Rac1 and RhoA in PBMCs from 1G and uG (25 hours total culture), n=7, donors are male (25 years old), and females (38, 46, 25, 27, 26 and 40 years old). Two-tailed paired t test is used to calculate P values. Data are plotted as mean ± SEM and source data are provided with the paper.
(E) G-LISA levels of active GTP-bound Rac1 and RhoA in PBMCs treated with TLR7/8 agonist (9 hours) from 1G and uG (total culture, 25 hours), n=7, donors are male (25 years old), and females (38, 46, 25, 27, 26 and 40 years old). One outlier from 1G Stim is removed individually from Rac1 and RhoA based on Grubb's (alpha=0.01) test. Two-tailed paired t test is used to calculate P values. Data are plotted as mean ± SEM and source data are provided with the paper.

Supplementary Figure 19 (related to Figure 6)

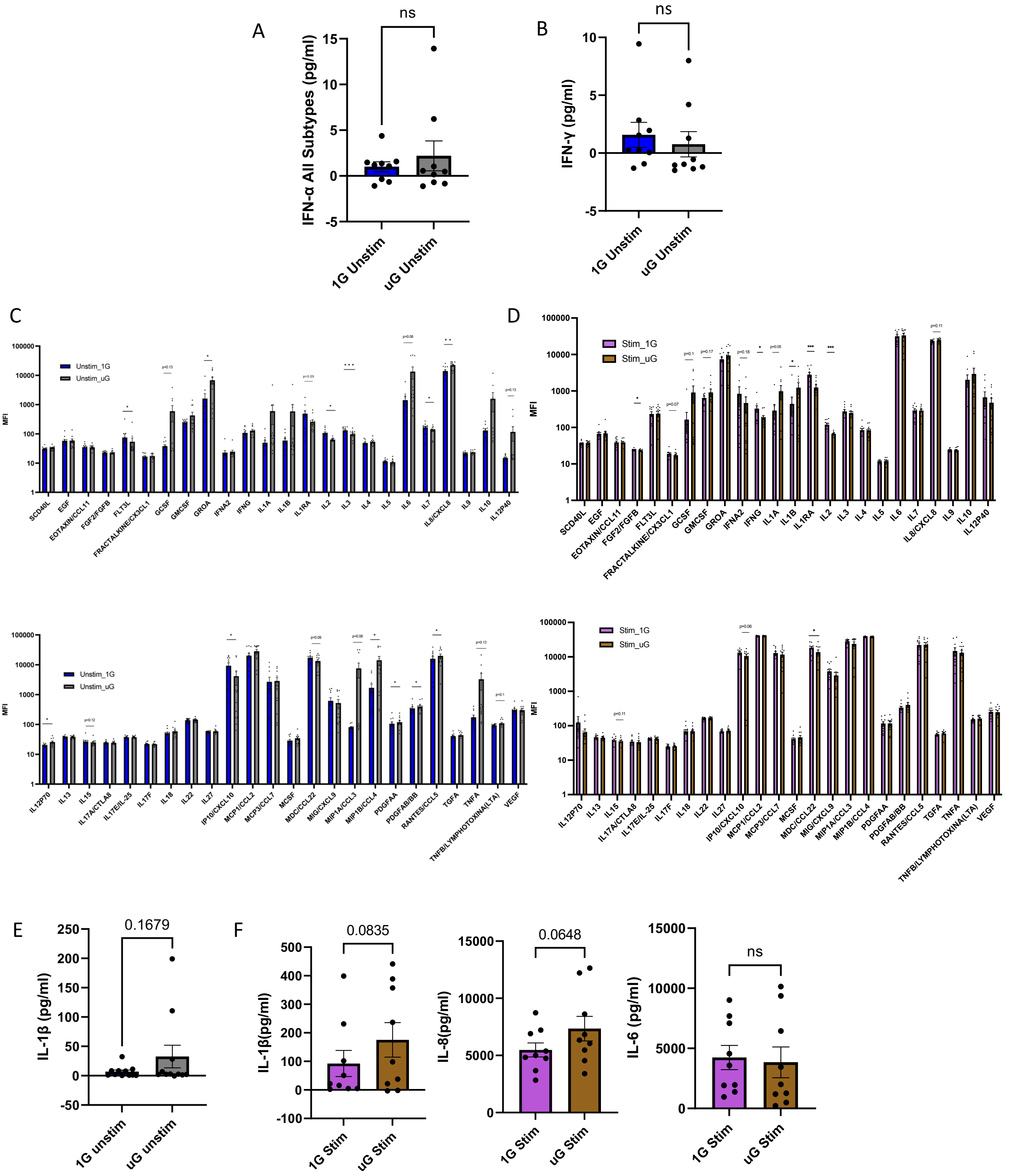

Supplementary Fig 19: Functional validation of the impact of simulated microgravity on overall immune cell cytokine production.

(A-B) ELISA validation results of cytokines IFN-α (A) and IFN-γ (B) in supernatants from unstimulated PBMCs after 25 hours 1G or uG treatment. n=9, 1 male (36 yrs) and 8 females (33, 25, 38, 46, 27, 25, 26, and 40 yrs). Two-tailed paired t test is used to calculate P values. Data are plotted as mean ± SEM and source data are provided with the paper.

(C-D) Luminex assay on cytokines secreted by unstimulated (C) and R848 stimulated (9 hours) (D) PBMCs after 25 hours 1G or uG treatment. n=12 for Unstimulated, and n=11 for Stimulated. 3 males (36, 33, 26 yrs) and 9 females (32, 25, 38, 46, 25, 27, 26, 40, 33 yrs) were used in Unstimulated. 2 males (36, 26 yrs) and 9 females (32, 25, 38, 46, 25, 27, 26, 40, 33 yrs) were used in Stimulated. Paired two-tailed t test is used to calculate P values, ***$P \leq 0.001$, **$P \leq 0.01$, *$P \leq 0.05$. Data are plotted as mean ± SEM and source data are provided with the paper.

(E-F) ELISA validation results of cytokines (E) IL-1β in unstimulated PBMCs, as well as (F) IL-1β, and IL-8, IL-6 in stimulated PBMCs (9 hours) after 25 hours 1G or uG treatment. n=11 for IL-1β unstim, donors were female (32, 25, 38, 46, 25, 27, 26, 40 years old) and male (36, 33, 26 years old). n=9 for the rest, 1 male (26 yrs) and 8 females (32, 25, 36, 46, 25, 27, 26, 40 yrs).

Two-tailed paired t test is used to calculate P values. Data are plotted as mean ± SEM and source data are provided with the paper.

Supplementary Figure 20

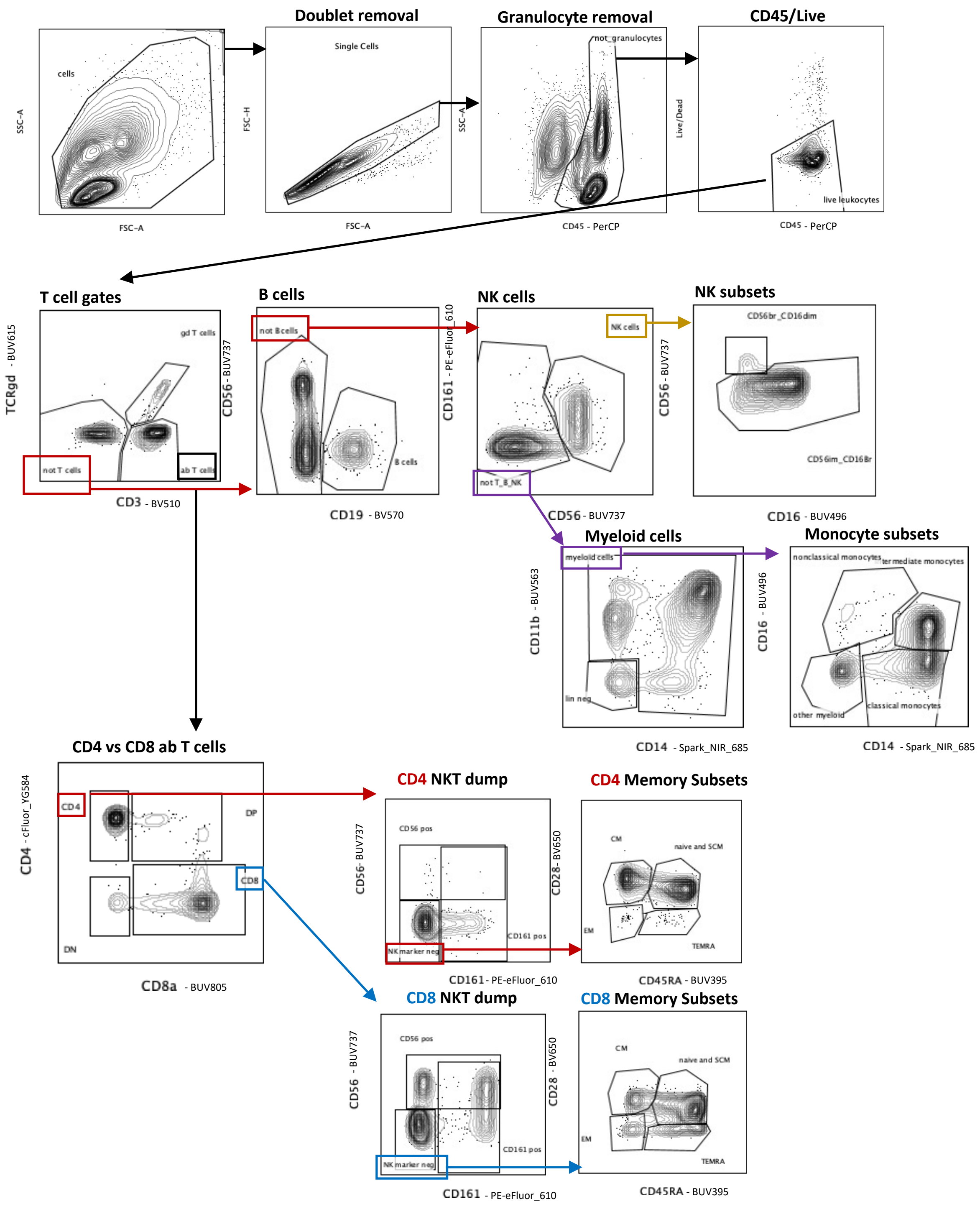

Supplementary Fig. 20: Gating strategy for flow cytometry immunophenotyping on PBMCs of 25-hour simulated microgravity treated with 9-hour R848 (1uM) in the presence of 2.5ug/mL Brefeldin A. NK, Natural killer; CM, Central memory; EM, Effector memory, SCM, Stem cell memory; TEMRA, CD45RA^{+} T effector memory.

Supplementary Figure 21

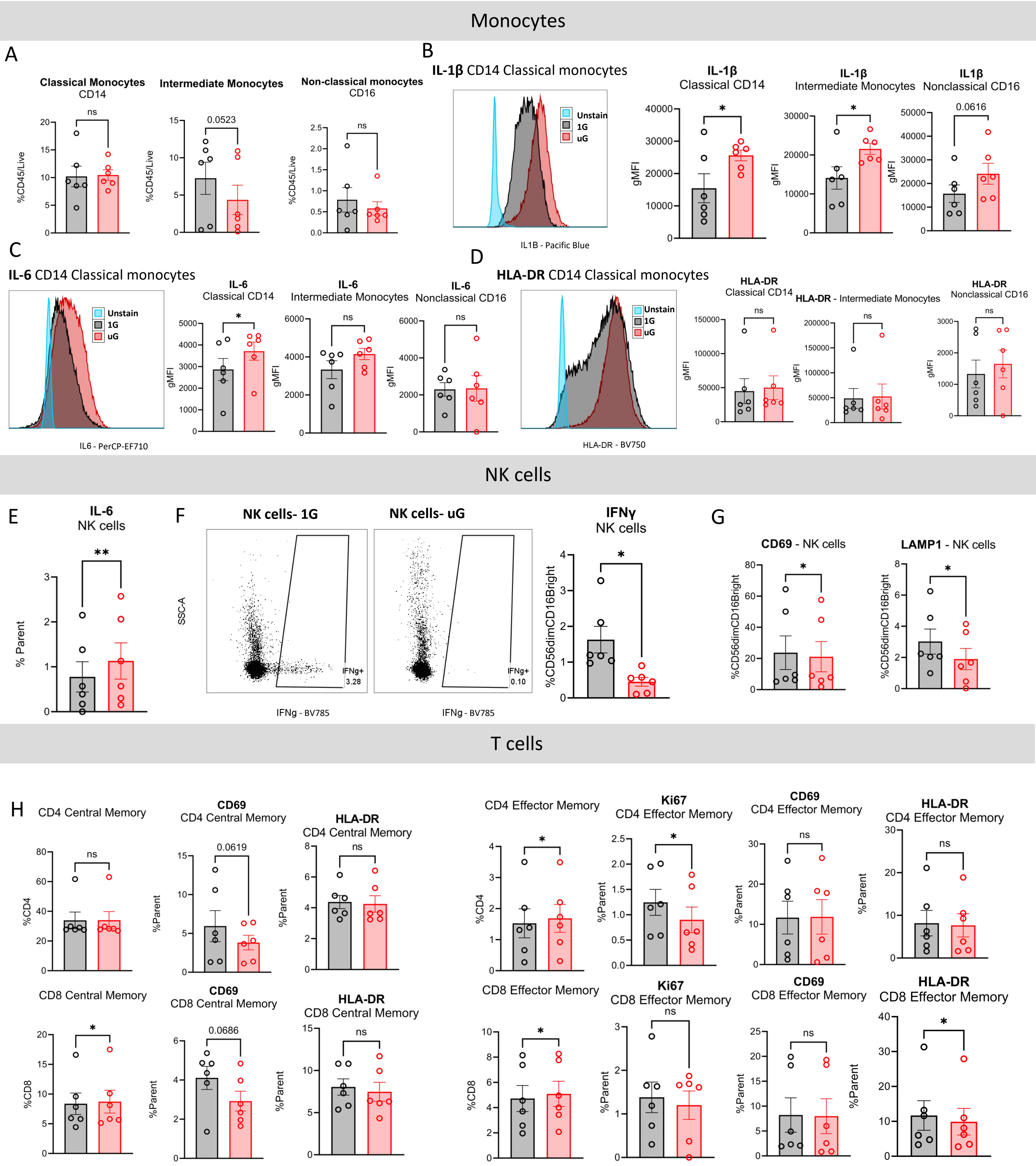

Supplementary Fig. 21: Flow cytometry analysis from PBMCs subjected to 1G (grey) and simulated uG (red) for 16 hours acclimation + 9 hours stimulation with R848 (1uM) in the presence of 2.5ug/mL Brefeldin A.
(A-D) The relative proportion of different monocytes were compared in (A), as well as comparison in the relative expression of IL-1β (B), IL-6 (C) and HLA-DR (D) in different monocyte subsets.
(E-G) The relative expression of (E) IL-6, (F) IFNγ, (G) CD69 and LAMP1 in CD56 dim CD16 bright NK cells were compared.
(H) The relative number of subsets of CD4 and CD8 T cells were compared, as well as the relative expression of CD69, HLA-DR, and Ki67 in effector memory cells.
Data were generated from n=6, males (40, 42, 43 yrs), females (38, 43, 35 yrs). All P-values were calculated from pair wise one-tailed t test comparisons, $*P \leq 0.05$. Data are plotted as mean ± SEM and source data are provided with the paper.
