# Supplementary material for: Single-cell analysis identifies conserved features of immune dysfunction in simulated microgravity and spaceflight
Source: Nat Commun. 2024 Jun 11;15:4795. doi: 10.1038/s41467-023-42013-y (PMC11166937; doi:10.1038/s41467-023-42013-y)
Supplement: Supplementary file 3 — Description of Additional Supplementary Files [file 41467_2023_42013_MOESM3_ESM.pdf]

Description of Additional Supplementary Files

Supplementary Data 1: A complete list of overall gene expression changes uG vs 1G (25 hours culture) in unstimulated peripheral blood mononuclear cells (PBMCs).

Supplementary Data 2: IPA pathways results of uG vs 1G unstimulated PBMCs (overall and individual cell types).

Supplementary Data 3: Differentially expressed genes (DEGs) of uG vs 1G in unstimulated PBMCs for each cell types.

Supplementary Data 4: A complete list of overall gene expression changes of uG vs 1G in R848 stimulated PBMCs (9 hours stimulation, 25 hours culture).

Supplementary Data 5: IPA pathways results of uG vs 1G stimulated PBMCs (overall and individual cell types).

Supplementary Data 6: DEGs of uG vs 1G in stimulated PBMCs for each cell types.

Supplementary Data 7: DEGs of stimulated and unstimulated PBMCs in 1G and uG conditions.

Supplementary Data 8: Conserved DEGs of uG vs 1G PBMCs under both stimulated and unstimulated conditions.

Supplementary Data 9: A complete list of overlapping genes between stimulated vs unstimulated in 1G and uG.

Supplementary Data 10: Complete lists of DEGs between uG vs 1G in both male and female and in both stimulated and unstimulated PBMCs. Data were generated from 1 female and 1 male donors.

Supplementary Data 11: CIBERSORTx results of cell type frequency of uG and 1G from 6 Bulk RNA-seq samples, containing different interventions (1G, uG, 1G + quercetin, uG + quercetin). Data were generated from 3 female and 3 male donors.

Supplementary Data 12: A complete list of DEGs of uG vs 1G generated by Bulk RNAseq validation, with and without quercetin treatment. Data were generated from 3 female and 3 male donors.

Supplementary Data 13: 210 DEGs overlapped between single-cell RNA-seq PBMCs (SC) and the I4 study (I4), using gene expression across all cells.

Supplementary Data 14: Pathway comparison of SC and I4.

Supplementary Data 15: Complete lists of DEGs of SC and the Twins Study (Twin).

Supplementary Data 16: Pathway comparison of SC and Twin.

Supplementary Data 17: GCEA compound repurposing results mapping transcriptional signatures to simulated uG.

Supplementary Data 18: Pathway comparison results of SC, bulk RNA-seq, and GLDS420 groups.

Supplementary Data 19: Antibody panels used for high-dimensional phenotyping of PBMCs by flow cytometry.
