# Supplementary material for: Single-cell analysis identifies conserved features of immune dysfunction in simulated microgravity and spaceflight
Source: Nat Commun. 2024 Jun 11;15:4795. doi: 10.1038/s41467-023-42013-y (PMC11166937; doi:10.1038/s41467-023-42013-y)
Supplement: Supplementary file 23 — Reporting Summary [file 41467_2023_42013_MOESM23_ESM.pdf]

nature portfolio

Corresponding author(s): Christopher E. Mason, David Furman, Daniel A. Winer

Last updated by author(s): 2023/08/23

Reporting Summary

Nature Portfolio wishes to improve the reproducibility of the work that we publish. This form provides structure for consistency and transparency in reporting. For further information on Nature Portfolio policies, see our Editorial Policies and the Editorial Policy Checklist.

Statistics

For all statistical analyses, confirm that the following items are present in the figure legend, table legend, main text, or Methods section.

n/a	Confirmed	
☐	☒	The exact sample size (*n*) for each experimental group/condition, given as a discrete number and unit of measurement
☐	☒	A statement on whether measurements were taken from distinct samples or whether the same sample was measured repeatedly
☐	☒	The statistical test(s) used AND whether they are one- or two-sided<br>*Only common tests should be described solely by name; describe more complex techniques in the Methods section.*
☐	☒	A description of all covariates tested
☐	☒	A description of any assumptions or corrections, such as tests of normality and adjustment for multiple comparisons
☐	☒	A full description of the statistical parameters including central tendency (e.g. means) or other basic estimates (e.g. regression coefficient) AND variation (e.g. standard deviation) or associated estimates of uncertainty (e.g. confidence intervals)
☐	☒	For null hypothesis testing, the test statistic (e.g. *F*, *t*, *r*) with confidence intervals, effect sizes, degrees of freedom and *P* value noted<br>*Give P values as exact values whenever suitable.*
☒	☐	For Bayesian analysis, information on the choice of priors and Markov chain Monte Carlo settings
☒	☐	For hierarchical and complex designs, identification of the appropriate level for tests and full reporting of outcomes
☐	☒	Estimates of effect sizes (e.g. Cohen's *d*, Pearson's *r*), indicating how they were calculated

Our web collection on statistics for biologists contains articles on many of the points above.

Software and code

Policy information about availability of computer code

Data collection	Image acquisition was performed using ZEN Microscopy Software (Zeiss). Flow cytometry data was collected using SpectroFlo (Cytek).
Data analysis	As detailed in the methods, the code used to analyze the sequencing data is available at GitHub repository (https://github.com/FEI38750/Immune_Dysfunction_in_Microgravity).<br><br>Single-cell RNA-seq analyses were performed in R (version 4.2.0), primarily using the Seurat R package (version 4.1.1) and custom analysis scripts. First, we executed a quality control step that removed the cells containing >10% mitochondrial RNA and <=250 genes/features. The doublet cells were identified and removed from the downstream analysis by using the DoubletFinder R package (version 2.0.3) 61 with parameters PCs=1:30, pN=0.25, and nExp=7.5%. To avoid the influence of hemoglobin transcripts on the analysis, we filtered out the putative red blood cells (defined by the method below) before the following process. Raw RNA counts were first normalized and stabilized with the SCTransform v2 function (SCT), then followed by the CCA integration workflow for joint analysis of single-cell datasets. In doing so, the top 3,000 highly variable genes/features among the datasets were used to run SCT; and then 3,000 highly variable genes/features and the 30 top principal components (PCs) with k.anchor=5 were used to find "anchors" for integration. The clustering step was executed by using the 30 top PCs summarizing the RNA expression of each cell with a resolution parameter of 0.8. To identify putative cell types, Azimuth (version 0.3.2) pipeline was used with the reference dataset of Human - PBMC celltype.l2. Cell type annotation results from Azimuth were validated by checking the markers of each of cell type (Supplementary Table 13). Gene differential expression analyses were done by Seurat PrepSCTFindMarkers then FindAllMarkers/FindMarkers functions with MAST algorithm. The pseudo-bulk analysis was conducted to find overall DEGs of uG against 1G in either unstimulated or stimulated PBMCs, using the FindMarker function with parameter min.pct=0.005 and logFC=0.1. To compare the stimulated and unstimulated PBMCs under uG and 1G conditions, we subtracted log2FC values of their DEGs (uG - 1G). The top 50 most upregulated DEGs between stimulated and unstimulated PBMCs under 1G were used for comparison. FindConservedMarkers function was used to find DEGs that are conserved between the groups with the same parameter settings as

FinderMarkers. The top 50 conserved DEGs specifically sensitive to uG were selected based on the rank of the absolute sum of log2FC values, derived separately from the sum of positive log2FC values and the sum of negative log2FC values. Rank-Rank Hypergeometric Overlap (RRHO) analysis was performed by using RRHO2 R package (version 1.0) to compare the differential expression patterns between 1G and uG of stimulated vs unstimulated PBMCs. The ranks of the genes in the two gene lists were determined by calculating -log10(adj.pvalue)*log2FC. Following differential expression, Ingenuity Pathway Analysis (IPA, Qiagen) was used to discover changes in enriched pathways in each comparison. DEGs with p-values < 0.05 and |Log2FC|> 0.1 were incorporated into the IPA canonical pathway analysis. To study the inferred trajectory of PBMC differentiation, cell trajectory analysis was performed by using the Monocle 3 R package (version 1.2.9). We first subsetted Seurat data to uG and 1G groups then run the functions as.cell_data_set(), cluster_cells(), and learn_graph(). Then, we ran order_cells() with the selection of cell types representing early development stages (CD4 naive, B naive, plasmablast, and HSPC) as the roots of the trajectory. The inflammatory aging (iAge) index was calculated by the sum of the cell scores that count by multiplying normalized and scaled gene expression with the corresponding coefficient of the gene in the iAge gene set. Cellular senescence was scored using Seurat AddModuleScore function on the SenMayo gene set. To study the difference in intercellular communication from APCs to T cells between uG and 1G, we used nichenetr R packages (version 1.1.0) to analyze cells in the dataset belonging to APCs (B cells, DCs, or monocytes) and T cell types. The "Differential NicheNet" workflow was implemented. The expressed genes in sender cells - APCs were selected if they were expressed in at least 10% of that APC cell population. The gene set of interest in receiver cells – T cells was defined by adjusted p-value <=0.05 and Log2FC >= 0.25 in the DEGs. Top 30 ligands that were further used to predict activated target genes and construct an activated ligand-receptor network. Default settings were used for all other parameters.

For bulk RNA sequencing, FASTQ raw reads were analyzed using the MTD pipeline. Differential gene expression analysis between groups was done by DESeq2 R package (version 1.36.0) with controlling for the subject effect. Genes with adjusted p-value<0.05 and |log2(FoldChange)| >0 were considered as differentially expressed. DEGs with p-values < 0.05 and |Log2FC|>0.5 were used for the IPA canonical pathway analysis. Different from single-cell (SC), in order to calculate the iAge index for bulk RNA-seq, normalized and scaled gene expression was multiplied with the gene's coefficient in the iAge gene set, then summed for each sample. Cellular senescence was scored using the ssGSEA method on the SenMayo gene set. Mouse spleen Bulk RNA-seq raw data was acquired from NASA GeneLab Data Repository with the accession ID GLDS-420. The detailed study description and experiment protocols are on the data repository https://genelab-data.ndc.nasa.gov/genelab/accession/GLDS-420. MTD pipeline was used to process the FASTQ raw data, generate the count matrix, and then analyze differentially expressed genes between Flight and Ground groups. Cell Type Frequency Changes within PBMCs were predicted by CIBERSORTx Docker image - Fractions Mode version 1.0. Our single-cell RNA-seq data from PBMCs was used to build the Signature Matrix File as the reference to predict the cell proportion in the bulk RNA-seq data.

For the viral and microbial abundance analysis, the output reads counts from MTD pipeline were then combined with the host reads and analyzed in R with Seurat package and other customized scripts. The relative abundance (frequency) of a virus or microbe was determined by dividing its reads count by the total reads count (host and non-host) in that sample. The classification results were further validated by using a different method Magic-BLAST.

The p-value of gene overlapping between two datasets was calculated by Fisher's Exact Test in GeneOverlap R package (version 1.32.0). The 375 DEGs in uG vs. 1G from unstimulated PBMCs single-cell RNA-seq results were used to match with the genes from PBMC bulk RNA-seq, i4, Twins, or JAXA studies. For the mouse genes in GLDS-420, we first converted them to the human orthologous before the analysis. In the matched genes, those expressions were in the same log2FC direction as 375 DEGs as well as with p-value < 0.05, were considered overlapping (except for i4, where either direction was considered overlapping). Complete linkage hierarchical clustering was used to analyze dissimilarities in genes or pathways between datasets, and the results were visualized by the ComplexHeatmap R package (version 2.12.0). Moreover, the IPA canonical pathway analysis was performed on the matched genes of I4 and Twins studies. The 106 core gene set was constituted by DEGs that consistently change their log2FC directions in both SC and bulk data of PBMCs. The alteration of the core gene set by the compound was measured by Gene Set Enrichment Analysis (GSEA) and Pearson correlation test.

Compound Analysis: FDA-approved drugs (n = 1692) are selected from the DrugBank database and food compounds (n = 7962) are selected from the FoodDB database as previously described (https://www.nature.com/articles/s41598-019-45349-y). LINCS compounds (n = 5414) are obtained from LINCS L1000 project. 'Compound' is used as a general term for 'drug', 'food compound' and 'LINCS compound' throughout the document. Compound-protein interactions are extracted from the STITCH database v5.0 by matching the InChI keys of drugs/food/LINCS compounds. STITCH collects information from multiple sources and individual scores from each source are combined into an overall confidence score. After processing, three data sets are obtained: i) drug-gene interaction dataset containing 1890 drugs and 16,654 genes with 542,577 interactions ii) food compound - gene interaction dataset containing 7654 compounds and 116,375 genes and 818,737 interactions iii) LINCS compound - gene interaction dataset containing 5414 compounds and 16,794 genes and 692,152 interactions. Statistical significance for the overlap between compound genes and the DEGs from the uG vs 1G of the unstimulated PBMCs single-cell RNA-seq is calculated using Fisher's exact test. The universal gene set contains all genes that interact with at least one compound. The compound with low p-value interacts with a higher proportion of the DEGs than that expected by chance. Statistically significant compounds were then obtained after Bonferroni adjustment of p-values. The pipeline for this compound analysis is implemented in the R script GCEA. Code for GCEA is available upon request, and provided to the editor during the submission of the manuscript.

Inspiration4 mission data: Four astronauts' transcriptomic data from the Inspiration4 (i4) mission was shared by Dr. Christopher E. Mason and his team at Cornell University. Blood samples were collected before (preflight L-92, L-44, and L-3), during, and after (R+1) the 3-day spaceflight in the SpaceX Dragon capsule. For this mission, we made use of data provided to us from the samples of the four astronauts comparing postflight (R+1) vs preflight (L-44) DEGs. Referring to the analysis workflow used by i4 Cornell team, a list of fold change and p values based on post flight vs preflight findings was generated. The Seurat FindMarker parameters used to calculate i4 DEGs were the same as those used for our 375 DEGs. Next, the DEGs and pathway overlap from i4 single-cell analysis were calculated by using the methods described above in the section on Gene set overlapping analysis.

2D 2-dimensional image analysis: Staining intensities, mitochondrial size, and punctate over diffuse index (defined as variance over mean) were determined in Image Analyst MKII 4.1.14 (Image Analyst Software, Novato, CA) in maximum intensity projection images using a custom pipeline. Cellpose 2.0 with the "cyto2" neural network was used for finding cells in the images based on nuclear and actin staining. Protruding actin bundles were analyzed by first binarizing projection images of actin using the trainable LABKIT segmentation, and this was followed by separation of protrusions and measurement of their maximal distance from the bulk of the cell using morphological erosion and distance image functions in Image Analyst MKII. Rescaled projection images were saved and further analyzed in CellProfiler 4.2.4, where images were segmented for nuclei and these segments were extended to the cell boundaries based on the phalloidin staining. These profiles were used for

measuring shape, granularity spectrum, and texture in actin, mitochondria, and nuclei. For actin granularity spectrum measurement the following parameters were used in CellProfiler MeasureGranularity function: "Subsampling factor for granularity measurements"=1, "Subsampling factor for background reduction=0.125", "Radius of structuring element"=12, "Range of granular spectrum" =16. Similar results were obtained using a set of discrete Fourier transformation-based Butterworth band pass filters in Image Analyst MKII for analysis of actin granularity spectrum changes in simulated microgravity and TLR stimulation. Here a series of 16 adjacent 4-pixels wide (in Fourier space of a 512×512 pixels image), 300-order band pass filters with "Corrected Integral" normalization and absolute value calculation were used starting at 1 pixel, and mean pixel intensities over whole cells in the filtered images were normalized to the unfiltered image. We have previously shown that this technique is primarily sensitive to sub-resolution changes in thickness of underlying filamentous structures, such as actin bundles in this case. We found no changes in granularity spectra measured by CellProfiler or Image Analyst MKII when analyzing mitochondria or nuclei of the same cells, excluding optical biases.

3D 3-dimensional image analysis: Mitochondria:cell volume fraction was measured using a modification of the "Mitochondria:cell volume fractionator (basic)" pipeline in Image Analyst MKII, using the hole-filled actin image as cell marker and MitoTracker Red as mitochondrial marker, and all image planes to measure areas of mitochondrial and cell profiles. Cell and nucleus volumes and surface areas were measured using Imaris 9.9 (Oxford Instruments, Concord, MA) using the Cell and Batch modules.
For 2D and 3D image analyses, tabular data generated by Image Analyst MKII, CellProfiler and Imaris were matched to conditions in Microsoft Excel and in Mathematica 13 (Wolfram Research, Champaign, IL) and visualized in Prism 9 (GraphPad, La Jolla, CA) for statistical analysis. Two-tailed Welch's t-test is used for all comparisons.

Statistical Analyses: In addition to the methods described above, the Wilcoxon Rank Sum Test was used to assess whether the distributions of data from cell score or microbiome abundance were significantly different between the 1G and uG cell populations from single-cell data. The association between single-cell and bulk RNA-seq in gene expressions was tested by Spearman's correlation. Mann-Whitney test was performed on ROS reduction by quercetin. Unpaired parametric two-tailed t-tests were performed on single-cell iAge, SenMayo and microbial abundance and imaging analyses for statistics. G-LISA, ELISA, Luminex and DCFDA results were assessed by paired parametric two-tailed t-test. However, given our existing transcriptomic and cytokine data showed decreased interferon coupled to increased IL-1β, IL-6, and IL-8 production in microgravity, for validation the flow cytometry results were assessed by one-tail paired parametric t-test. In PBMCs bulk RNA-seq results, the difference in iAge and SenMayo scores of samples with or without compound treatment was evaluated by two-tailed paired parametric t-test. R (version 4.2.0) and GraphPad Prism 9 were used to conduct the statistical analyses. Significance was set at 0.05. Outliers in datasets were assessed using Grubbs' test (alpha =0.01) and specified in figure legends if any were removed for all data.

For manuscripts utilizing custom algorithms or software that are central to the research but not yet described in published literature, software must be made available to editors and reviewers. We strongly encourage code deposition in a community repository (e.g. GitHub). See the Nature Portfolio guidelines for submitting code & software for further information.

Data

Policy information about availability of data

All manuscripts must include a data availability statement. This statement should provide the following information, where applicable:

- Accession codes, unique identifiers, or web links for publicly available datasets
- A description of any restrictions on data availability
- For clinical datasets or third party data, please ensure that the statement adheres to our policy

Raw and processed 10x Genomics and bulk RNA-seq data can be found at Gene Expression Omnibus (GEO) using accession number GSE218937 [https://www.ncbi.nlm.nih.gov/geo/query/acc.cgi?acc=GSE218937]. Mouse spleen bulk RNA-seq fastq files were downloaded directly from the NASA GeneLab website: https://genelab-data.ndc.nasa.gov/genelab/accession/GLDS-420. I4, Twin study, and JAXA data were provided directly by our collaborators, as described in the method section. Source data are provided in the paper. The code used for analysis of sequencing data is available at GitHub and Zenodo repository (https://github.com/FEI38750/Immune_Dysfunction_in_Microgravity; DOI: https://zenodo.org/record/8247816). The remaining data and codes are available either within the Article, Supplementary Information, or Source Data file.

Human research participants

Policy information about studies involving human research participants and Sex and Gender in Research.

Reporting on sex and gender	Blood (buffy coats) was purchased from Stanford Blood Center (SBC) under signed contract in an anonymous fashion and thus this work is not considered a clinical trial with human research participants. Where available, sex of the blood donor is listed in the figure legend of the manuscript.
Population characteristics	Information was provided by the SBC on age and sex on each donor as listed in the figure legends of the manuscript. Donor ages span from 20 to 46 years. In total, 14 female and 13 male donors were used in this study. The selection of donor sex was not predefined; it was exclusively determined based on donor availability at the SBC. The purchaser of the blood (Buck Institute) has no access to the blood donor's identification information.
Recruitment	N/A - no clinical trial with human research participants for the simulated microgravity work. Recruitment information for I4, Twins study and JAXA study are provided in their respective primary manuscripts.
Ethics oversight	Material (human buffy coats) was obtained from the Stanford Blood Center under official signed contract agreement with the Buck Institute, following the Stanford Blood Center's Certification of Human Subjects Approval for minimal risk research related activities (IRB eProtocol#: 13942). Human data on I4, Twin study and JAXA were provided directly from the respective institutes. These institutes obtained the IRB approvals and they are listed in their respective primary manuscripts.

Note that full information on the approval of the study protocol must also be provided in the manuscript.

Field-specific reporting

Please select the one below that is the best fit for your research. If you are not sure, read the appropriate sections before making your selection.

- [x] Life sciences
- [ ] Behavioural & social sciences
- [ ] Ecological, evolutionary & environmental sciences

For a reference copy of the document with all sections, see nature.com/documents/nr-reporting-summary-flat.pdf

Life sciences study design

All studies must disclose on these points even when the disclosure is negative.

Sample size	For single-cell RNA seq, we analyzed PBMCs from 2 individuals (1 male and 1 female) and sequenced a total of 55,648 PBMCs, which gave us a comprehensive integration of blood immune cell populations, even on rare cell subsets. Furthermore, we used additional 25 donors to validate the single-cell data with Bulk RNA-seq, imaging, and protein level assessment including GLISA, ELISA, Luminex and Flow cytometry. We also had access to 3 astronaut space-flight datasets and 1 mouse space-flight data to validate our findings, giving us strong validation power.
Data exclusions	Outliers in datasets were assessed using the Grubbs' statistical outlier test and specified in the figure legends when removed.
Replication	Data in this paper were validated in multiple validation models, for instance single-cell sequencing was validated by additional 6 samples of bulk RNA-seq as well as using data from multiple space-flight samples and other techniques including microscopy. Drug targets identified by machine learning in this paper were also validated by bulk RNA-seq. In each set of in vitro validation experiments encompassing GLISA, ELISA, imaging, and flow cytometry, we used samples from distinct donors. To bolster reproducibility, a total of 27 independent donor samples were used.
Randomization	Single cell samples included one young male and one young female, both CMV+. The remaining samples, such as for validation studies, were used in the order they were received, with age, and sex being randomly incorporated based on material available at the SBC.
Blinding	Samples were used randomly in the order they were received. Lab members were not blinded as to which samples were subject to 1G vs uG when performing most validation studies, except that super-resolution microscopy scoring was performed blindly using computer algorithms.

Reporting for specific materials, systems and methods

We require information from authors about some types of materials, experimental systems and methods used in many studies. Here, indicate whether each material, system or method listed is relevant to your study. If you are not sure if a list item applies to your research, read the appropriate section before selecting a response.

Materials & experimental systems

n/a	Involved in the study	
[ ]	[x]	Antibodies
[x]	[ ]	Eukaryotic cell lines
[x]	[ ]	Palaeontology and archaeology
[x]	[ ]	Animals and other organisms
[x]	[ ]	Clinical data
[x]	[ ]	Dual use research of concern

Methods

n/a	Involved in the study	
[x]	[ ]	ChIP-seq
[ ]	[x]	Flow cytometry
[x]	[ ]	MRI-based neuroimaging

Antibodies

Antibodies used

G-LISA detection kit: Cytoskeleton Inc., Denver, CO. (GTP-bound Cdc42 BK127-S; GTP-bound Rac1 BK128-S ; GTP-bound RhoA BK124-S)
ELISA detection kit: ELISA IFN-α all subtypes (cat# 41135; Pestka Biomedical Laboratories, Inc., Piscataway, NJ). ELISA IFN-γ (cat# 430104; Biolegend Inc., San Diego, CA). ELISA IL-8 (cat# 431504; Biolegend Inc., San Diego, CA), ELISA IL-6 (cat# 430504; Biolegend Inc., San Diego, CA), ELISA IL-1β (cat# 437004; Biolegend Inc., San Diego, CA)

Flow cytometry immunophenotyping:
CD45RA BUV395 5H9 BD_Biosciences 740315 1.25ul/test
CD16 BUV496 3G8 BD_Biosciences 612944 1ul/test
CD161 PE-eflour_610 hp-3g10 Invitrogen 61-1619-42 5ul/test
TCRγδ BUV615 11F2 BD_Biosciences 751308 5ul/test
CD56 BUV737 NCAM16.2 BD_Biosciences 564447 1.25ul/test
CD8 BUV805 SK1 BD_Biosciences 612889 1.25ul/test
IgD BV480 IA6-2 BD_Biosciences 566138 1.25ul/test

CD3 BV510 SK7 BioLegend 344828 4ul/test
CD19 BV570 HIB19 Biolegend 302236 5ul/test
CD28 BV650 CD28.2 BioLegend 302945 5ul/test
HLA-DR BV750 L243 Biolegend 307671 5ul/test
CD45 PerCP 2D1 BioLegend 368506 3.75ul/test
CD4 cFluor_YG584 SK3 CYTEK R7-20041-100T 1.25ul/test
CD25 PE-Fire_700 M-A251 BioLegend 356145 5ul/test
CD95 PE-Cy5 DX2 BioLegend 305610 1.25ul/test
CD27 APC-H7 M-T271 BD_Biosciences 560222 2.5ul/test
CD14 Spark_NIR_685 63D3 Biolegend 367150 2.5ul/test
CD38 APC-Fire_810 HB7 BioLegend 356643 1.5ul/test
CD69 APC FN50 BioLegend 310909 1.25ul/test
CD11b BUV563 M1/70 BD_Biosciences 741242 0.5ul/test
CD24 BV711 ML5 BioLegend 311135 2.5ul/test
LAMP1 PE-Cy7 H4A3 BioLegend 328617 2.5ul/test
IL6 PerCP-EF710 MQ2-13A5 Invitrogen 46-7069-42 0.25ul/test
IL1-b Pacific Blue H1b-98 BioLegend 511709 1ul/test
ki-67 BV605 Ki-67 BioLegend 350522 5ul/test
IFNg BV785 4S.B3 Biolegend 502541 1.75ul/test

Validation

References are listed in vendors' websites:
CD45RA BUV395 BD_Biosciences 740315 https://www.bdbiosciences.com/en-us/products/reagents/flow-cytometry-reagents/research-reagents/single-color-antibodies-ruo/buv395-mouse-anti-human-cd45ra.740315
CD16 BUV496 BD_Biosciences 612944 https://www.bdbiosciences.com/en-us/products/reagents/flow-cytometry-reagents/research-reagents/single-color-antibodies-ruo/buv496-mouse-anti-human-cd16.612944
CD161 PE-eflour_610 Invitrogen 61-1619-42 https://www.thermofisher.com/antibody/product/CD161-Antibody-clone-HP-3G10-Monoclonal/61-1619-42
TCRγδ BUV615 BD_Biosciences 751308 https://www.bdbiosciences.com/en-us/products/reagents/flow-cytometry-reagents/research-reagents/single-color-antibodies-ruo/buv615-mouse-anti-human-tcr.751308
CD56 BUV737 BD_Biosciences 564447 https://www.bdbiosciences.com/en-us/products/reagents/flow-cytometry-reagents/research-reagents/single-color-antibodies-ruo/buv737-mouse-anti-human-cd56.612766
CD8 BUV805 BD_Biosciences 612889 https://www.bdbiosciences.com/en-us/products/reagents/flow-cytometry-reagents/research-reagents/single-color-antibodies-ruo/buv805-mouse-anti-human-cd8.612889
IgD BV480 BD_Biosciences 566138 https://www.bdbiosciences.com/en-us/products/reagents/flow-cytometry-reagents/research-reagents/single-color-antibodies-ruo/bv480-mouse-anti-human-igd.566138
CD3 BV510 BioLegend 344828 https://www.biolegend.com/en-us/products/brilliant-violet-510-anti-human-cd3-antibody-9880?GroupID=BLG5900
CD19 BV570 Biolegend 302236 https://www.biolegend.com/en-us/products/brilliant-violet-570-anti-human-cd19-antibody-7367
CD28 BV650 BioLegend 302945 https://www.biolegend.com/en-us/products/brilliant-violet-650-anti-human-cd28-antibody-13271
HLA-DR L243 Biolegend 307671 https://www.biolegend.com/en-us/products/brilliant-violet-750-anti-human-hla-dr-antibody-17450
CD45 PerCP BioLegend 368506 https://www.biolegend.com/en-us/products/percp-anti-human-cd45-antibody-12393
CD4 cFluor_YG584 CYTEK R7-20041-100T https://cytekbio.com/products/cfluor-568-anti-human-cd4?variant=32351881986084
CD25 PE-Fire_700 BioLegend 356145 https://www.biolegend.com/en-us/products/pefire-700-anti-human-cd25-antibody-19788
CD95 PE-Cy5 BioLegend 305610 https://www.biolegend.com/en-us/products/pe-cyanine5-anti-human-cd95-fas-antibody-644
CD27 APC-H7 BD_Biosciences 560222 https://www.bdbiosciences.com/en-us/products/reagents/flow-cytometry-reagents/research-reagents/single-color-antibodies-ruo/apc-h7-mouse-anti-human-cd27.560222
CD14 Spark_NIR_685 Biolegend 367150 https://www.biolegend.com/en-us/products/spark-nir-685-anti-human-cd14-antibody-18625
CD38 APC-Fire_810 BioLegend 356643 https://www.biolegend.com/en-us/products/apc-fire-810-anti-human-cd38-antibody-19549
CD69 APC BioLegend 310909 https://www.biolegend.com/en-us/products/apc-anti-human-cd69-antibody-1674
CD11b BUV563 BD_Biosciences 741242 https://www.bdbiosciences.com/en-us/products/reagents/flow-cytometry-reagents/research-reagents/single-color-antibodies-ruo/buv563-rat-anti-cd11b.741242
CD24 BV711 BioLegend 311135 https://www.biolegend.com/en-us/products/brilliant-violet-711-anti-human-cd24-antibody-12508
LAMP1 PE-Cy7 BioLegend 328617 https://www.biolegend.com/en-us/products/pe-cyanine7-anti-human-cd107a-lamp-1-antibody-7707
IL6 PerCP-EF710 Invitrogen 46-7069-42 https://www.thermofisher.com/antibody/product/IL-6-Antibody-clone-MQ2-13A5-Monoclonal/46-7069-42
IL1-b Pacific Blue BioLegend 511709 https://www.biolegend.com/en-us/products/pacific-blue-anti-human-il-1beta-antibody-5836
ki-67 BV605 BioLegend 350522 https://www.biolegend.com/en-us/products/brilliant-violet-605-anti-human-ki-67-antibody-8708
IFNg BV785 Biolegend 502541 https://www.biolegend.com/en-us/products/brilliant-violet-785-anti-human-ifn-gamma-antibody-7986

Flow Cytometry

Plots

Confirm that:

- [x] The axis labels state the marker and fluorochrome used (e.g. CD4-FITC).
- [x] The axis scales are clearly visible. Include numbers along axes only for bottom left plot of group (a 'group' is an analysis of identical markers).
- [x] All plots are contour plots with outliers or pseudocolor plots.
- [x] A numerical value for number of cells or percentage (with statistics) is provided.

Methodology

Sample preparation	De-identified peripheral blood buffy coat samples were obtained from healthy human donors between the ages of 20 and 46 from the Stanford University Blood Center. PBMCs were isolated using a Ficoll gradient method. PBMCs were counted and re-suspended in complete media at 1x10e6 cells/ml (RPMI 1640, 10% Fetal Bovine Serum, 2mM L-Glutamine, 1% penicillin/ streptomycin, 0.1mM non-essential Amino acid,1mM sodium pyruvate, 50uM 2-mercaptoethanol,10mM HEPES). To stimulate PBMCs, samples were mixed with 1uM R848 (TLR7/8 agonist) and 2.5ug/ml Brefeldin A after 16 hours, for 9 hours of stimulation. At the end of each experiment, the cell suspension was quickly collected, spun down at 500x g, washed twice with FACS buffer, and stained with LIVE/DEAD™ Fixable Blue Dead Cell Stain kit for viability followed by Fc-blocking with human igG. The cells were further stained with fluorophore-conjugated surface antibodies for 20 min at 4C and intracellular antibodies for 30 min at room temperature following fixation and permeabilization using Foxp3 staining buffer set.
Instrument	Cell phenotyping was analyzed on a Cytek Aurora™ instrument.
Software	The analysis was done using FlowJo™.
Cell population abundance	We did not perform any sorting. The abundance of live leukocytes post surface and intracellular staining was above 91% of all leukocytes across all samples.
Gating strategy	The first gate we used was SSC-A/FSC-A to select non-debris. We then performed a single-cell gate with FSC-H/FSC-A and selected cells on the diagonal. Often, we cannot obtain blood immediately after it was drawn, which can result in granulocyte contamination even after Ficoll separation in our PBMCs. For that reason, we drew a gate using SSC-A/CD45 to select only CD45+ high leukocytes. We further selected live leukocytes by gating cells with low to zero Live/Dead stains. After gating for live leukocytes, we used TCRgd/CD3 to differentiate ab T cells (CD3+), gd T cells (TCRgd+), and non-T cells (CD3-, TCRgd-). We then gated ab T cells with CD4/CD8a to differentiate CD8+ and CD4+ T cells. Both CD4+ and CD8+ T cells were further refined using CD56/CD161 to deselect the ones with NK markers, and then further gated into subsets with CD28/CD45RA. Effector memory cells express low CD45RA and CD28, while central memory cells express low CD45RA and high CD28. The non-T cells were gated for CD19 expression to remove B cells that express CD19. The non-T and B cell population was then separated using CD56 expression, where NK cells were selected for further subtyping. CD56/CD16 were used to subset NK cells into CD56 bright and CD16 dim vs CD56 dim and CD16 bright. The latter subset was assessed for cytokine and activation marker production. The non-T, B, and NK population was filtered using CD11B/CD14, discarding CD11B and CD14 low cells to obtain the myeloid cell population. Lastly, the myeloid cells were compartmentalized into classical myeloid cells that express CD14, intermediate myeloid cells that express both CD14 and CD16, non-classical myeloid cells that express CD16, and other myeloid cells that are double negative for CD14 and CD16. See Supplementary Figure 20.

- [x] Tick this box to confirm that a figure exemplifying the gating strategy is provided in the Supplementary Information.
